# Direct Regenerating Cathode Materials from Spent Lithium‐Ion Batteries

**DOI:** 10.1002/advs.202304425

**Published:** 2023-11-13

**Authors:** Yuanqi Lan, Xinke Li, Guangmin Zhou, Wenjiao Yao, Hui‐Ming Cheng, Yongbing Tang

**Affiliations:** ^1^ Advanced Energy Storage Technology Research Center Shenzhen Institute of Advanced Technology Chinese Academy of Sciences Shenzhen 518055 China; ^2^ Shenzhen College of Advanced Technology University of Chinese Academy of Sciences Shenzhen 518055 China; ^3^ Nano Science and Technology Institute University of Science and Technology of China Suzhou 215123 China; ^4^ Shenzhen Geim Graphene Center Tsinghua Shenzhen International Graduate School Tsinghua University Shenzhen 518055 China; ^5^ Shenzhen Key Laboratory of Energy Materials for Carbon Neutrality Shenzhen 518055 China; ^6^ Faculty of Materials Science and Energy Engineering/Institute of Technology for Carbon Neutrality Shenzhen Institute of Advanced Technology Chinese Academy of Sciences Shenzhen Shenzhen 518055 P. R. China

**Keywords:** direct regeneration, methodologies, perspectives, repair, spent lithium‐ion battery cathode

## Abstract

Recycling cathode materials from spent lithium‐ion batteries (LIBs) is critical to a sustainable society as it will relief valuable but scarce recourse crises and reduce environment burdens simultaneously. Different from conventional hydrometallurgical and pyrometallurgical recycling methods, direct regeneration relies on non‐destructive cathode‐to‐cathode mode, and therefore, more time and energy‐saving along with an increased economic return and reduced CO_2_ footprint. This review retrospects the history of direct regeneration and discusses state‐of‐the‐art development. The reported methods, including high‐temperature solid‐state, hydrothermal/ionothermal, molten salt thermochemistry, and electrochemical method, are comparatively introduced, targeting at illustrating their underlying regeneration mechanism and applicability. Further, representative repairing and upcycling studies on wide‐applied cathodes, including LiCoO_2_ (LCO), ternary oxides, LiFePO_4_ (LFP), and LiMn_2_O_4_ (LMO), are presented, with an emphasis on milestone cases. Despite these achievements, there remain several critical issues that shall be addressed before the commercialization of the mentioned direct regeneration methods.

## Graphical abstract

Direct regeneration method takes a shortcut to obtain cathodes with performance near to, same as, or even superb to that of the cathode synthesized from the normal routes, without the disruption and reformation of crystal structure. Here is an overview of directly regenerating cathode materials from spent lithium‐ion batteries and a summary of representative works in this field.

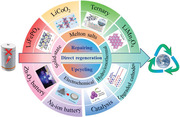

## 1 Introduction

**Figure 1:**
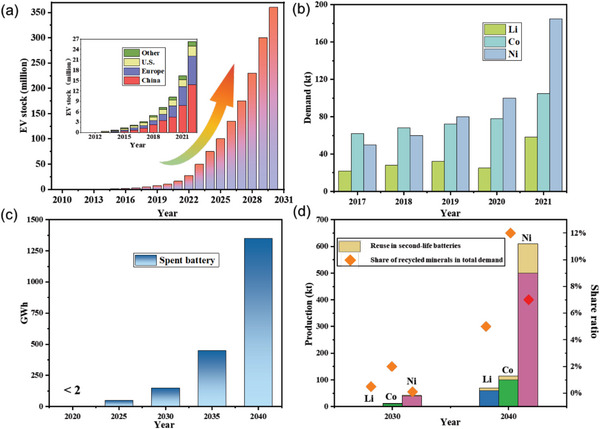
The expanding market of LIBs. a) The EV stock in main economic entities from 2010 to 2022 and the estimated total EV stock in the next 10 years under Net Zero Emission by 2050 scenario; b) The demand for Li, Co, and Ni from 2017 to 2021 to fabricate LIBs; c) The estimated amounts of spent batteries in the next 20 years; d) The estimated production of Li, Co, and Ni from spent batteries and their corresponding ratios of total supply requirements in 2030 and 2040. Reproduced with permission.^[^ ^2^, ^4^, ^6^ ^]^ Copyright 2021–2023, IEA.

Replacing fuel‐driven vehicles with electric vehicles (EV) is regarded as an important movement to achieve carbon neutrality and a sustainable society.^[^ ^1^ ^]^ As a result, the EV market, including battery electric vehicles (BEV) and plug‐in hybrid electric vehicles (PHEV), has seen a sharp increase during the last few years. According to the reports from the International Energy Agency (IEA),^[^ ^2^ ^]^ the global EV stock reached up to 27 million in 2022, among which China and Europe jointly contributed to a majority of global EV sales (85%). Under the Net Zero Emission by 2050 scenario, the EV stock is estimated to reach over 350 million in 2030 (**Figure** **1**a). Meanwhile, it can be anticipated that the number of retired EVs will be continuously growing in the coming years.

Lithium‐ion batteries (LIBs) are the sole energy storage and conversion device in current on‐road EVs. Mimic to the EVs market, the LIBs market is experiencing quick growth.^[^ ^3^ ^]^ Therefore, the demand for critical minerals to fabricate LIBs, especially lithium, cobalt, and nickel, has been dramatically increasing (Figure 1b), and its expanding demand is foreseeable. Meanwhile, the avalanche of end‐of‐life (EOL) LIBs is approaching with the increase of retired EVs (Figure 1c). The untreated EOL LIBs are not only a waste of valuable resources but also will cause environmental issues. In contrast, the recycled production of Li, Co, and Ni from spent batteries may compensate for the resource shortage, accounting for up to 12% (60 kt), 7% (100 kt), and 5% (500 kt) of total supply requirements in 2040 for cobalt, nickel, and lithium, respectively (Figure 1d),^[^ ^4^ ^]^ under the sustainable development scenario. Therefore, recycling the retired LIBs in a green way paves the way for the sustainable improvement of the LIBs industry and our society.^[^ ^5^ ^]^

**Figure 2:**
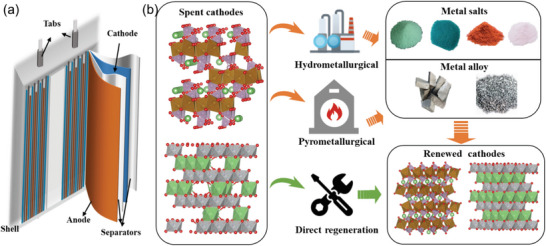
Recycling processes of spent cathode materials. a) The illustration of a typical LIB; b) The illustration of recycling procedures of hydrometallurgical, pyrometallurgical, and direct regeneration methods.

A typical LIB contains negative and positive electrodes, separators, electrolytes, and shells as shown in **Figure** **2**a. The valuable metals, including Li, Co, and Ni, mostly exist in the cathodes. Hence, the recycling of cathodes from spent batteries is of vital importance. The whole process for recycling spent LIBs consists of pretreatment and recycling. The aim of pretreatment is to separate the different parts of LIBs safely and effectively. The pretreatment process concludes with discharge, the dismantling of retired batteries, and the separation of different components. The recycling of other critical materials has been detailly summarized in ref. [7] and will not be discussed in this review. The mainstreamed methods of spent LIB cathode recycling include hydrometallurgical, pyrometallurgical, and direct regeneration methods.^[^ ^8^ ^]^ The commonality between the hydrometallurgical method based on acid leaching and the pyrometallurgical method based on high‐temperature calcination lies in the complete destroy of the crystal structure and lattice rearrangement,^[^ ^9^ ^]^ forming metal salts and alloys, respectively (Figure 2b). The recycled products are then used in the existing cathode material‐producing line to synthesize corresponding new cathode materials. On the contrary, the direct regeneration method takes a shortcut to obtain cathodes with performance near to, same as, or even superb to that of the cathode synthesized from the normal routes, without the disruption and reformation of the pristine crystal structures of the cathode. Thus, the energy consumption is lower than the hydrometallurgical and pyrometallurgical methods.^[^ ^10^ ^]^

**Figure 3:**
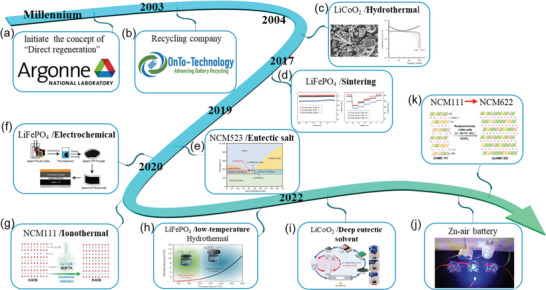
The developing history of direct regeneration and the representative works in this field. a) Initiation. b) The first relevant company. c–k) Representative works of direct repairing or upcycling spent cathode. Reproduced with permission.^[^ ^13^ ^]^ Copyright 2004, Elsevier. Reproduced with permission.^[^ ^14^ ^]^ Copyright 2017, The Royal Society of Chemistry. Reproduced with permission.^[^ ^15^ ^]^ Copyright 2019, Wiley‐VCH. Reproduced with permission.^[^ ^16^ ^]^ Copyright 2020, The Royal Society of Chemistry. Reproduced with permission.^[^ ^17a^ ^]^ Copyright 2020, Wiley‐VCH. Reproduced with permission.^[^ ^17b^ ^]^ Copyright 2020, Elsevier. Reproduced with permission.^[^ ^17c^ ^]^ Copyright 2022, Oxford Academic. Copyright 2020, Elsevier. Reproduced with permission.^[^ ^18a^ ^]^ Copyright 2022, Elsevier. Copyright 2020, Elsevier. Reproduced with permission.^[^ ^18b^ ^]^ Copyright 2022, Proceedings of the National Academy of Sciences.

The concept of direct regeneration was initiated by researchers from Argonne National Laboratory (ANL) around the millennium (**Figure** **3**a). More specifically, Linda Gaines, a transportation systems analyst from ANL proposed the concepts of “direct regeneration” and “cascade utilization” of used LIBs.^[^ ^11^ ^]^ Relevant reports are rare at the beginning and have become prosperous only in recent years. In 2003, Onto‐Technology, a recycling company, was founded that had relevant patents^[^ ^12^ ^]^ (Figure 3b). 1 year later, Kim et al. first reported the regeneration of LiCoO_2_ (LCO) by a hydrothermal method (Figure 3c).^[^ ^13^ ^]^ Along with the retirement of LiFePO_4_ (LFP) batteries, Song et al. applied a simple sintering method to repair degraded LFP cathode (Figure 3d).^[^ ^14^ ^]^ In 2019, a eutectic salt method was proposed for the ternary system that greatly reduced the repairing temperature (Figure 3e).^[^ ^15^ ^]^ Nearly at the same time, an electrochemical method was designed to repair LFP by pairing directly with a pre‐lithiated anode (Figure 3f).^[^ ^16^ ^]^ Afterward, optimized methods were advocated to use lower temperatures (Figure 3g–i).^[^ ^17^ ^]^ The expanded usage of degraded cathode was reported around 2020 (Figure 3j,k).^[^ ^18^ ^]^

So far, the concept of direct regeneration consists of two aspects. One is to repair the cathode so that it can exhibit a nearly to or the same electrochemical performance as that of the pristine cathodes. The other is the upcycling of the cathode, which enable the regenerated cathode to exhibit superior electrochemical performance to that of the pristine one or utilize in other application. This review addresses both aspects, focusing on the history and the state‐of‐the‐art development in direct regeneration. Closely‐related methodologies are introduced first and their pros‐and‐cons are discussed. Milestone studies on repairing and upcycling are in‐depth analyzed. The remaining issues and challenges are thereafter prospected.

## 2 Methodology

**Figure 4:**
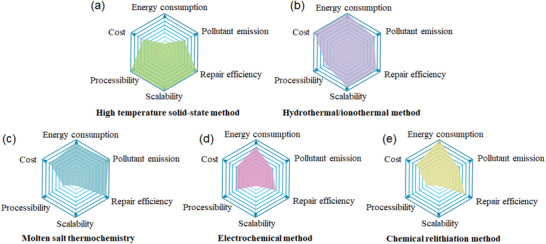
Comparison of different direct regeneration methods in terms of energy consumption, pollutant emission, repair efficiency, scalability, processability, and cost, with the degree of superiority represented by the line segments of the hexagon from the center to each of the six vertices. a) High‐temperature solid‐state method; b) Hydrothermal/ionothermal relithiation; c) Melton salt thermochemistry; d) Electrochemical method; e) Chemical relithiation method.

The critical point in the direct regeneration of cathodes from spent LIBs is to restore the crystalline structure of the cathode without completely disrupting the chemical bonds of the cathodes.^[^ ^5^, ^19^ ^]^ Several methods have been proposed for regenerating spent cathodes by targeting the primary degradation mechanism of cathode materials. In general, Li vacancy defects are common in cycled cathodes.^[^ ^20^ ^]^ Besides, other degradation mechanisms within the cycled cathodes, such as the oxidation of transition metal cation accompanied by Li loss,^[^ ^17b^ ^]^ anti‐site defects, irreversible phase transformation,^[^ ^21^ ^]^ and particle cracking,^[^ ^22^ ^]^ also need to be addressed. In this case, the introduction of energy and compensation of elements into the repair systems becomes the main idea to directly recover the spent cathodes. Until now, various methods of direct cathode repair have been proposed including high‐temperature solid‐state method, hydrothermal and ionothermal relithiation, molten salt thermochemistry, electrochemical method, and chemical relithiation method. **Figure** **4** evaluates them from aspects of energy consumption, pollutant emissions, repair efficiency, scalability, processability, and cost. Details of evaluation information are provided in the Supporting Information. It is shown that the high‐temperature solid‐state method, as a mature material preparation method, owns the merits of high repair efficiency, scalability, and processability. Yet its energy consumption is relatively high due to the required high temperature. Other methods, including hydrothermal/ionothermal relithiation, molten salt thermochemistry, and electrochemical methods, show the superiority in energy consumption. Nonetheless, short‐annealing often serves as an inevitable post‐treatment process to obtain the final repaired product with good crystallinity. Furthermore, based on the methods for repairing spent cathodes, improved procedures have been proposed to achieve better performances or for other applications, i.e., the upcycling of the spent cathodes. In this section, we will give a brief summary of methodologies of repairing and upcycling of spent cathodes and discuss the pros and cons of each.

### 2.1 High‐Temperature Solid‐State Method

High‐temperature solid‐state processes are the most common methods to directly repair spent cathode materials, with the features of high‐temperature and dry regeneration. Heat drives the compensate elements diffusing to their original position under the thermodynamics condition, thus restoring the crystal structure of the materials. Besides, the cracks of the cathode particle fussed under high temperatures. The high‐temperature solid‐state method with its brute force has been widely applied in this research field. In this process, relithiation and tuning the valence of the transition metal can be achieved by adding Li resources and controlling the atmosphere of heat treatment, respectively, and simultaneously.

Based on the sole high‐temperature solid‐state method, Chen et al. explored the rational temperature range and atmosphere for repairing spent LFP cathode.^[^ ^23^ ^]^ It is demonstrated that high temperatures (600–800 °C) and a reductive atmosphere are essential to resynthesize the LFP from decomposed compounds. Fan et al. reported a simple sintering method to repair the degraded LiNi_0.5_Co_0.2_Mn_0.3_O_2_ (NCM523) under the temperature of 800 °C for 8 h in the air.^[^ ^24^ ^]^ The high‐temperature solid‐state method has also been applied to repair spent LCO under the temperature of 900 °C in air, in which Li_2_CO_3_ worked as a replenishing reagent.^[^ ^25^ ^]^ Similarly, other reported high‐temperature solid‐state methods require additional Li resources for better cathode repair.^[^ ^26^ ^]^ It is worth mentioning that elemental quantitation characterization, such as the inductively coupled plasma‐optical emission spectrometry (ICP‐OES) technique, is required to guide the exact amount of the additional Li element.

Improved procedures based on high‐temperature solid‐state methods for repairing spent cathodes have been proposed. A solid phase method promoted by activated carbon nanotubes (CNTs) was carried out by Song et al.^[^ ^26a^ ^]^ The activated CNTs provided reducibility from Fe^3+^ to Fe^2+^ and decreased the impedance of the repaired cathode. Thus, the repaired LFP cathode exhibited excellent long‐term cycling stability in the half‐cell test. In another work, two‐stage calcination in an oxygen atmosphere was proposed for directly repairing the NCM523 cathode.^[^ ^26c^ ^]^ Through this high‐temperature solid‐state method, the Li deficiencies of the spent NCM523 cathode were replenished by the Li_2_CO_3_ surface occupants formed after electrochemical cycling. Furthermore, the rack salt type NiO phase was transformed into a layered phase after calcination. The oxygen atmosphere not only provided an oxidizing condition for the phase transformation but also hindered the release of oxygen from the cathode, thus recovering the well‐ordered layered α‐NaFeO_2_ phase.

Elemental doping and coating are commonly used to improve the electrochemical performance of cathode materials. Accordingly, they were introduced into the upcycling of the spent cathodes. For instance, Jia et al. utilized the spent NCM523 as a Ni/Mn doping precursor to regenerate a high‐voltage LCO from the retired LCO cathode.^[^ ^27^ ^]^ The spent NCM523 was pretreated to be the Ni/Mn/Co oxides mixture by leaching and precipitating. Then, the obtained mixture was grounded with spent LCO and extra Li_2_CO_3_. After calcination at 900 °C for 10 h, the upcycled high‐voltage LCO was prepared, which can cycle stably up to 4.6 V. Doping can also be realized by pre‐mixing with certain reagents before high‐temperature solid‐state calcination, as proved in the upcycling of spent LFP,^[^ ^28^ ^]^ LCO,^[^ ^29^ ^]^ etc. Besides, layer coating on the cathode has been achieved through a high‐temperature solid‐state method recently. Gao et al. proposed a coating strategy to recover the spent LCO cathode by adding Al_2_O_3_ and ball milling before high‐temperature calcination.^[^ ^30^ ^]^ Similarly, interface engineering by coating Li_1.4_Al_0.4_Ti_1.6_(PO_4_)_3_ was utilized to regenerate high‐voltage LCO from retired cathodes.^[^ ^31^ ^]^ Overall, the synthesis idea of doping or coating is to introduce additional reagents into the spent cathodes before high‐temperature calcination.

In general, the high‐temperature solid‐state method as a mature technology in material science has been widely applied in the direct regeneration of spent cathodes. To some extent, the high‐temperature solid‐state method is irreplaceable owing to the unique effect of heat, such as fusing the microcracking of particles^[^ ^32^ ^]^ and increasing the crystallinity of materials,^[^ ^33^ ^]^ compared with other available techniques. Moreover, it is usually an essential post‐treatment for other relithiation treatments such as hydrothermal and molten salt methods.

### 2.2 Hydrothermal/Ionothermal Relithiation

Mass diffusion between solid interfaces is mostly difficult and often requires a high temperature to conquer the energy barrier. In a hydrothermal system, water acts as a superior mass and heat transferor. Hence, targeting the Li deficiency, the common defect within the retired cathode materials, the hydrothermal method has been introduced to relithiate the spent cathodes at a relatively lower temperature. Unlike the high‐temperature solid‐state method, hydrothermal relithiation does not acquire a precise quantitation of additional Li source and is superior in energy consumption.

**Figure 5:**
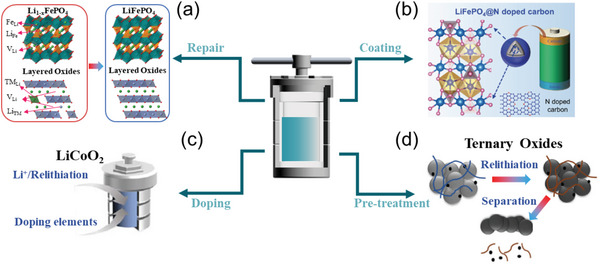
Schematic illustration of the functions of the hydrothermal method. a) Repairing. b) Coating. Reproduced with permission.^[^ ^34b^ ^]^ Copyright 2022, Wiley‐VCH. c) Doping. Reproduced with permission.^[^ ^36^ ^]^ Copyright 2022, Wiley‐VCH. d) One‐step pretreatment‐and‐repairing. Reproduced with permission.^[^ ^34c^ ^]^ Copyright 2022, Wiley‐VCH.

Hydrothermal relithiation has been applied for direct repairing spent LFP, ternary transition cathodes, LCO, and LiMn_2_O_4_ (LMO).^[^ ^34^ ^]^ For example, Shi et al. proposed a non‐destructive approach to direct repair spent LCO cathode by hydrothermal relithiation method and subsequent short‐annealing.^[^ ^34a^ ^]^ In the hydrothermal relithiation step, the spent LCO cathode was placed in a Teflon‐lined autoclave containing 4 m lithium hydroxide (LiOH) solution at 220 °C for 4 h. After relithiation, the crystallinity of the cathode was further improved by the subsequent short annealing at 800 °C. This combined method of hydrothermal relithiation and short annealing was proven to be a simple and efficient approach to repair spent LCO, as illustrated in **Figure** **5**a. Recently, modified hydrothermal relithiation methods have been proposed. By adding a redox medium into the reaction system, a low‐temperature hydrothermal relithiation method at 100 °C was achieved to repair the spent ternary cathode.^[^ ^35^ ^]^ Green‐reducing agents (GAs) were added to the hydrothermal system to create a reductive environment. Hence, the activated barrier of Fe^3+^ to Fe^2+^ reduction was lowered and the Li insertion to the cathode was facilitated. As a result, the reaction pressure could be lowered to 1 bar, providing a safer operating condition. Similar works have also been reported for repairing spent LFP,^[^ ^17b^ ^]^ in which the usage of citric acid (CA) with a redox potential of approximately −0.34 V (vs standard hydrogen electrode, SHE) enables the relithiation temperature as low as 80 °C, showing the superiority of the GAs.

The hydrothermal method can also realize other functions including coating and element doping. By introducing an extra reagent (polyvinylpyrrolidone [PVP]) containing C and N elements,^[^ ^34b^ ^]^ the N‐doping carbon layer was homogeneously coated on the repaired LFP (Figure 5b), which achieved an excellent long‐term cycling performance in both half‐cell and full cell tests. By adding nickel and manganese acetates, Ni–Mn elements were co‐doped into the retired LCO (Figure 5c).^[^ ^36^ ^]^ Additionally, a one‐step pretreatment‐and‐relithiation was achieved by hydrothermal method^[^ ^34c^ ^]^ (Figure 5d). In detail, the PVDF binder was decomposed in the hydrothermal condition, thus the adhesion between active materials and current collectors was decreased. At the same time, the Li‐deficient cathode was relithiated.

In general, hydrothermal relithiation owns the merit of lower energy consumption, and the exact quantitation of additional Li source reagent is no longer mandatory. Its shortages lie in the need for a short annealing to reach better electrochemistry, and the unrecyclable solution. In that case, ionothermal relithiation was proposed to address the mentioned problems. Specifically, Wang et al. reported an ionothermal relithiation method without subsequent calcination,^[^ ^17a^ ^]^ which is superior to conventional hydrothermal relithiation. In this work, the LiBr and 1‐ethanol‐3‐methylimidazolium bis(trifluoromethanesulfonyl)imide ([C_2_OHmim][NTf_2_]) ionic liquid (IL) solution worked as an optimized precursor for relithiation of NCM523. The repaired NCM523 cathode showed almost the same capacity as the original NCM523 cathode. The IL provides a null vapor pressure due to the strong Coulombic interaction between the constituent ions.^[^ ^37^ ^]^ In addition, ILs are polar solvents that allow reasonably good solubility of inorganic precursors.^[^ ^38^ ^]^ Therefore, ionothermal methods are an ideal strategy for the relithiation of spent cathodes under relatively moderate conditions. Furthermore, the used ILs can be easily recycled and reused for the relithiation of spent cathodes. In this work, up to 98.7% of ILs can be recovered. Despite the high cost of ILs, it will be economically beneficial if the ILs could be recycled and reused.

### 2.3 Molten Salt Thermochemistry

Molten salts are homogeneous solutions when heated above the temperature of the molting point. Molten salts solutions are ideal reaction mediums for relithiating spent cathode materials, which provide fast mass transport. Among them, the eutectic salt mixture owns a lower melting temperature than that of any constituent salt at normal pressure, according to the phase diagrams. Hence, the thermochemical repair method utilizing molten salt, especially eutectic melting salt, has attracted the attention of researchers in recent years. Specifically, for Ni‐containing cathodes, a hydro‐free relithiation method is preferred to avoid the proton attack in an aqueous environment.^[^ ^10b^ ^]^ For instance, Shi et al. first reported ambient‐pressure relithiation of degraded NCM523 cathodes via eutectic Li^+^ molten‐salt solutions under a low operating temperature of 300 °C, which is much lower than the conventional high‐temperature solid‐state method.^[^ ^15^ ^]^ However, the solidified salts on the regenerated cathodes should be removed afterward. In this case, the regenerated cathode is washed with deionized water after the molten salt thermochemical treatment. As some cathode materials need a water‐free environment to prevent by‐products on the surface, subsequent short annealing is introduced to eliminate the negative effects of the washing step.

Based on molten salt thermochemistry, improved methods have been developed. For example, Deng et al. proposed a facile strategy to realize the relithiation and impurity removal of spent ternary cathode simultaneously via the molten salt method.^[^ ^39^ ^]^ In detail, LiNO_3_ was introduced into the molten salts, working as an oxidizing reagent to remove the remained carbon impurity. Liu et al. proposed an approach to repair spent LFP where the FeC_2_O_4_ was introduced, providing a reductive environment to suppress the Fe(II) oxidation.^[^ ^40^ ^]^ Wang et al. fabricated a Ni‐containing molten salts to upcycle the spent LiNi_1/3_Co_1/3_Mn_1/3_O_2_ (NCM111) to LiNi_0.6_Co_0.2_Mn_0.2_O_2_ (NCM622) by simultaneously Ni and Li insertion.^[^ ^18a^ ^]^

Deep eutectic solvents (DESs) have been widely acknowledged as a kind of near‐room temperature eutectic mixtures, which share many similar characteristics and properties with ILs.^[^ ^41^ ^]^ Wang et al. designed a LiCl–CH_4_N_2_O DES to repair the spent LCO cathode.^[^ ^17c^ ^]^ The operating temperature is 120 °C, lower than the ionothermal methods mentioned above, which is attributed to the significantly smaller absorption energy of Li^+^ by urea molecules than that of Co^2+^. Furthermore, the recycling of used molten salts was also realized in this work. In general, DES is a eutectic mixture of Lewis or Brønsted acids, which shows superiority in cost, biodegradability, and nontoxicity compared to ILs.^[^ ^42^ ^]^ DES‐based methods have great potential for direct regeneration of spent cathodes in an economically beneficial and green way.

In short conclusion, molten salt thermochemistry is gradually gaining attention for both repairing and upcycling spent cathode materials. This nonaqueous method is favored for the relithiation of Ni‐containing cathodes. Despite this, there remain other binary or ternary Li^+^ molten salt systems to be explored. Generally, decreasing the operating temperature and recycling the used molten salts are the main developing trends of this method.

### 2.4 Electrochemical Methods

Electrochemical methods refer to relithiate spent cathodes with the help of an external electric field. Universally, two main approaches have been reported to fabricate electrochemical relithiation systems. One is that the cycled positive electrode is assembled with a reference electrode and inert counter electrode, such as an Ag/AgCl electrode and Pt plate, immersed in a Li^+^‐containing aqueous solution.^[^ ^43^ ^]^ The other is to directly assemble with a pre‐lithiated negative electrode^[^ ^16^ ^]^ or separator.^[^ ^44^ ^]^ On the one hand, the former always requires post‐treatments after relithiation such as the removal of current collectors and purification of active materials. In addition, the proton attack in the aqueous solution may destroy the cathodes. On the other hand, the latter exhibits simplicity without complicated processes except for electrochemical activation, although the crystal elapses were not fully recovered. Moreover, the applicability to other cathodes except for LFP and LCO needs to be further verified.

In short conclusion, the electrochemical relithiation methods have not been widely accepted and applied, but the direct assembly with a pre‐lithiated negative electrode or separator shows a promising application due to its simplicity.

### 2.5 Chemical Relithiation Methods

**Figure 6:**
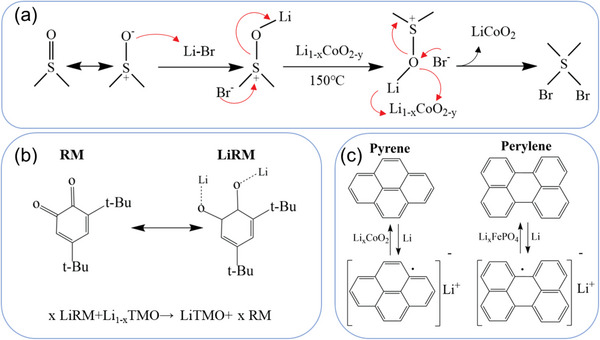
Scheme of the mechanism of several chemical relithiation methods. a) Auto‐oxidation relithiation mechanism. Reproduced with permission.^[^ ^45^ ^]^ Copyright 2022, Elsevier. b) Relithiation via a redox mediator. Reproduced with permission.^[^ ^46^ ^]^ Copyright 2021, American Chemical Society. c) Li^+^‐electron concerted radical redox process. Reproduced with permission.^[^ ^47^ ^]^ Copyright 2021, American Chemical Society.

In chemical relithiation methods, the organic chemicals with tunable potential worked as reducing reagents, accelerating the Li^+^ diffusion and the electron transferring from solution to cathodes. For example, Fei et al. proposed an auto‐oxidation strategy to repair spent LCO cathode, where the dimethyl sulfoxide (DMSO) worked as both solvent and oxygen donor.^[^ ^45^ ^]^ The relithiation mechanism is shown in **Figure** **6**a. DMSO with the cosolvent property works as a high charge flux medium to transport the Li^+^ and O^2−^, thus recovering spent cathode materials under a relatively low temperature of 150 °C. Park et al. proposed a relithiation method utilizing the quinone‐based redox mediators (RM) as Li transferors from the Li source to the spent cathode.^[^ ^46^ ^]^ Particularly, 3,5‐di‐tert‐butyl‐o‐benzoquinone (DTBQ) dissolved in dimethoxyethane (DME) showed the best relithiation performance, of which the relithiation mechanism is shown in Figure 6b. DTBQ in DEM took Li^+^ away from the Li metal surface and transferred Li^+^ to the spent cathode. It is worth noting that this relithiation method demonstrated its superior simplicity, just stirring at room temperature. In addition, the used DTBQ‐DME solution could be recycled and reused. Targeting the valence increase of transition metal ions in the spent cathodes, reductive lithiation agents were proposed to replenish Li^+^ in the spent cathodes through the redox reaction. Wu et al. developed a chemical relithiation method, where polycyclic aryl–lithium compounds served as both the reducing agent and Li^+^ donor to replenish the Li loss in degraded LFP and LCO cathodes.^[^ ^47^ ^]^ Specifically, pyrene with a redox potential of 0.82 V versus Li^+^/Li, which is above the decomposition potential of LFP (0.59 V vs Li^+^/Li) and below the potential of reversible Li^+^ intercalation reaction (3.42 V vs Li^+^/Li), was utilized to relithiate spent LFP cathode. Similarly, perylene demonstrated a perfect relithiation performance for the spent LCO cathode. It is worth noting that the reaction time was only 10 min, which is time‐saving and sheds light on its potential sustainable application in the industry.

In short, chemical relithiation methods based on redox reactions have demonstrated their simplicity, processability, and possibility of large‐scale production. However, there are still problems to be solved. For example, this method cannot fully recover the crystal distortion of spent cathodes, and subsequent heat treatment is required. Secondary air pollution may occur due to the use of volatile organic solvents. There remain other chemical relithiation systems to be explored for pursuing a green, economical, and high‐efficient way to recycle spent cathodes.

## 3 Achievements of Spent Cathode Repairing

The degraded LIBs cathodes include four main kinds, that is, LFP, ternary oxide, LCO, and LMO. Retired LFP and ternary oxides come mainly from the EOL EVs for now, and will also come from the echelon used battery products. As each EV or echelon‐used application field usually consumes a large amount of LIBs, recollected retired LFP and ternary oxides are often massive and more homogeneous. In contrast, retired LCO and LMO are mostly from smaller devices such as electronic devices or two‐wheeler vehicles. The recollected ones are often a mixture of different apparatuses with diverse life experiences and therefore shall be more diverse in both component and structure.

### 3.1 Direct Repair of LFP

Owning to the superiority of stability, safety, and low cost among multifarious cathode materials, LFP has captured a third of the market of EVs.^[^ ^48^ ^]^ With the booming of the EV market, the effective and environmentally‐friendly recycling and regeneration of LFP become urgent.^[^ ^49^ ^]^ Decades of studies disclose that the degradation of LFP cathode resulted mainly from Li inventory loss^[^ ^16^, ^50^ ^]^ and Li–Fe anti‐site defect.^[^ ^20a^ ^]^ Aimed to address these key issues, several methods have been proposed to repair LFP cathode including solid‐state methods,^[^ ^28^ ^]^ hydrothermal technique,^[^ ^34^, ^51^ ^]^ electrochemical relithiation,^[^ ^16^, ^44^ ^]^ chemical relithiation methods,^[^ ^47^ ^]^ molten salts methods,^[^ ^40^ ^]^ and innovative methods.^[^ ^52^ ^]^

High‐temperature solid‐state methods combined with adding extra Li resources have been proposed to recover the Li vacancy and anti‐site defects, which has proved to be of great potential. On the one hand, crystal defects can be recovered with heat treatment. On the other hand, Li atoms from extra Li‐containing reagents diffuse into the cathode and replenish the Li vacancy with the drive of heat. Under a reductive atmosphere, LFP was recovered from other phases (FePO_4_, Fe_2_O_3_, P_2_O_5_, and Li_3_PO_4_) through heat treatment, without using additional reagents.^[^ ^23^ ^]^ The element ratio of the used cathode can be determined by ICP‐OES, thus giving support to element compensation during heat‐treatment recovery.^[^ ^53^ ^]^ Recently, an effective solid phase method promoted by activated CNTs has been proposed to directly regenerate spent LFP.^[^ ^26a^ ^]^ The activated CNTs contain reductive hydroxyl and carboxyl, which accelerated the reduce of Fe^3+^ to Fe^2+^. Furthermore, CNTs remain during inert atmosphere sintering and significantly reduce the impedance of regenerated LFP. Meanwhile, Li_2_CO_3_ acts as a Li compensation reagent, thus replenishing the Li vacancy. In this work, the repaired LFP cathodes exhibited a discharge capacity of 155.47 mAh g^−1^ at 0.05 C with 650 °C heat treatment. In general, heat treatment has been widely applied to restore the crystal structure of spent cathode materials for its convenience.

**Figure 7:**
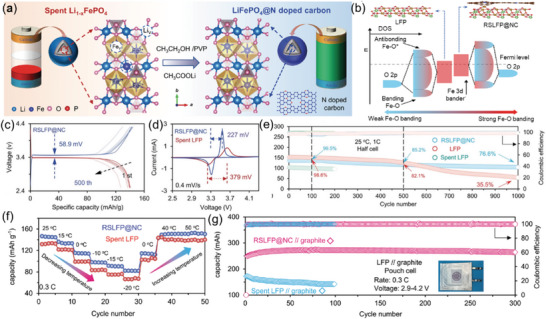
The repair of spent LFP via hydrothermal relithiation. a) Schematic of hydrothermal reparation for spent LFP cathode; b) Illustration of the rise of the d‐band center in RSLFP@NC; c) The Galvanostatic charge/discharge profiles of the RSLFP@NC at 1 C. d) CV curves of the spent LFP and RSLFP@NC. f) High‐ and low‐temperature of the spent LFP and RSLFP@NC. e,g) Long‐term cycling performances of RSLFP@NC half‐cell and full‐cell. Reproduced with permission.^[^ ^34b^ ^]^ Copyright 2022, Wiley‐VCH.

The hydrothermal method has been proved to be another effective method for repairing spent LFP cathodes.^[^ ^5^, ^54^ ^]^ Jia et al. proposed an environmentally friendly method that combines hydrothermal treatment and sintering to repair the spent LFP cathode.^[^ ^34b^ ^]^ This method aimed to elevate the d‐band center of Fe, thus stabilizing Fe ions in the lattice, limiting the formation of the anti‐site defects, and improving the long‐term cycling performance of the repaired LFP cathode. As shown in **Figure** **7**a, ethanol provided a reducing environment and CH_3_COOLi acted as a Li source. Most importantly, PVP was introduced into the hydrothermal system to form N‐doped carbon, thus constructing a heterogeneous interface in between (RSLFP@NC). Nitrogen elements with high electronegativity doped in the carbon strongly affect the electronic configuration near the Fermi energy for the Fe elements near the surface. More specifically, it raises the d‐band center of Fe and stabilizes the Fe─O bonding, as shown in Figure 7b. The specific discharge capacities of RSLFP@NC at the first and 500th cycles were 139.1 and 126.8 mAh g^−1^ at 1 C, respectively (Figure 7c). The voltage gap of two redox peaks of RSLFP@NC is smaller than that of spent LFP (Figure 7d), demonstrating the higher reversibility of Li extraction and insertion. The RSLFP@NC also exhibited superior temperature durability, as shown in (Figure 7f), reaching a reversible discharge capacity of 82.2 mAh g^−1^ at −20 °C. The long‐term cycling performance of RSLFP@NC has been greatly improved, demonstrating a 76.6% retention of initial capacity after 1000 charge–discharge cycles in half cell test (Figure 7e) and negligible capacity fade after 300 charge–discharge cycles in RSLFP@NC||graphite full cell test (Figure 7g).

**Figure 8:**
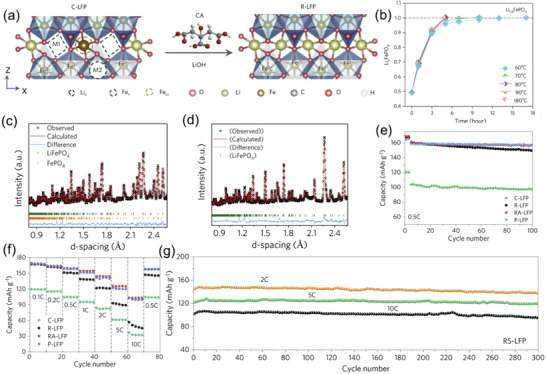
The repair of spent LFP via aqueous solution relithiation. a) The illustration of aqueous solution relithiation process for repairing cycled LFP; b) The evolution of LFP composition during relithiation at different temperatures; The Rietveld refinements of neutron diffraction of c) C‐LFP and d) R‐LFP; e) Cycling stability and f) rate performance of C‐LFP, R‐LFP, RA‐LFP, and P‐LFP; g) Long‐term cycling performance of RA‐LFP. Reproduced with permission.^[^ ^17b^ ^]^ Copyright 2020, Elsevier.

Extended from the hydrothermal method, an aqueous solution relithiation aimed at target healing has been proposed by Xu et al.^[^ ^17b^ ^]^ One of the spotlights is the replacement of pressured reactors, which is essential in hydrothermal methods, by low‐cost vessels without extra safety precautions. **Figure** **8**a illustrates the relithiation process. The key of the method is the use of CA, which provides a reductive environment and lowers the activation barrier for Fe migrating. Thus, the anti‐site defects could be restored. Furthermore, LiOH dissolved in the CA solution and worked as an extra Li source that replenished the Li vacancy. At a relatively low temperature (≈80 °C), the relithiation kinetics negligibly change compared with that at a higher temperature (Figure 8b), benefiting the efficiency and low energy consumption. As a result, the saturation vapor pressure of water at operating temperatures was low enough (<1 bar) to eliminate the need for pressurized reactors in this process. In addition, short annealing treatment for repaired LFP (RA‐LFP) further reduces the defects and improves the crystallinity. The Rietveld refinement patterns of neutron diffraction of cycled LFP (C‐LFP, Figure 8c) and repaired LFP (R‐LFP, Figure 8d) demonstrate the efficiency of this method. RA‐LFP exhibits a superior capacity up to 162, 144, and 102 mAh g^−1^ at 0.2, 2, and 10 C, respectively. In addition, RA‐LFP could cycle stably for 100 times, with negligible capacity loss, which is comparable to a pristine LFP cathode (Figure 8e). Compared to R‐LFP, RA‐LFP shows a better rate performance (Figure 8f), with an imperceptible capacity loss during high C‐rate cycling (Figure 8g). Overall, the Li vacancies and Li/Fe anti‐site defects of C‐LFP are healed by this aqueous relithiation method and a short annealing treatment.

For the electrochemical relithiation method, Wang et al. assembled the original spent LFP electrode with a pre‐lithiated graphite electrode without any further processing of the spent LFP, which simplified the recycling procedures.^[^ ^16^ ^]^ During the electrochemical process, spent LFP was relithiated and recovered directly. For the molten salt method, a low‐temperature lithium nitrate (LiNO_3_) molten salt process (300 °C) combined with carbon reduction has been proven efficient for regenerating spent LFP.^[^ ^40^ ^]^ LiNO_3_ worked as both molten salt media and Li source. Sucrose was used as a carbon source to provide a reductive environment. The collapsed and Li‐deficient structure was recovered after short molten‐salt relithiation and subsequent annealing. In addition, during the relithiation step, more (101) crystal planes were exposed, facilitating Li^+^ transportation. Hence, the repaired LFP cathode exhibited a better rate performance than the pristine LFP.

Furthermore, other direct repairing methods for spent LFP have been proposed. Recently, an innovative two‐step “ice & fire” method, a template‐assisted regeneration process that synergizes defect‐targeted healing and surface modification, has been introduced by Sun et al.^[^ ^52^ ^]^ Based on the aqueous solution relithiation method, the freeze‐drying step and annealing were introduced into the regeneration process. A unique 3D‐interconnected porous carbon network on the surface of LFP particles was formed by this method, thus increasing the electronic conductivity. Additionally, doping with N‐containing reagents before annealing, such as urea, further improves the electrochemical performance of the repaired LFP cathode, which exhibits a capacity of up to 169.74 and 141.79 mAh g^−1^ at 0.1 and 1 C, respectively.

In general, a reductive environment is often required for the repair of spent LFP cathode to both suppress the Fe(II) oxidation and reduce the Fe(III). Most of the mentioned methods are suitable for repairing spent LFP. It is worth noting that excessive Li replenishing reagents and prolonged reacting time will decompose LFP into Li_3_PO_4_,^[^ ^40^ ^]^ leading to decreasing productivity. Thus, the condition of the LFP repairing process should be carefully tuned. In preparation for the coming wave of large numbers of LFP‐based EVs being taken off the road, effective, low‐cost, and environmentally friendly recycling methods for direct regeneration of spent LFP need to be proposed, developed, and utilized.

### 3.2 Direct Repair of Ternary Cathodes

Layered ternary transition metal oxide cathodes with high energy densities have dominated the market of cathodes for EVs. Quite a few researches focusing on the direct repair of ternary cathodes have been published in recent years and have achieved impressive results. As summarized in **Table** **1**, the developed methods include direct solid‐state sintering treatment,^[^ ^24^, ^26^ ^]^ hydrothermal method combining with annealing,^[^ ^34^, ^35^ ^]^ molten salt,^[^ ^55^ ^]^ and ionothermal relithiation.^[^ ^17a^ ^]^ It is noticeable that most of the methods need a last‐step annealing process to further improve the crystallinity of cathodes. Until now, heat treatment is almost an unavoidable process for repairing cathodes.

**Table 1:** Summary of the works for direct repairing ternary cathodes.

Ternary cathodes	Direct repairing method	Half‐cell electrochemical performance	Full‐cell electrochemical performance	Ref.
NCM523	Direct high‐temperature solid‐state sintering treatment (800 °C, 8 h)	162.1 mAh g^−1^ (1st discharge, 2.8–4.3 V, 15 mA g^−1^); 94.52% retention after 100 cycles (2.8–4.3 V, 75 mA g^−1^)	NA	[24]
NCM111	Two‐step sintering treatment (500 °C, 2 h; 850 °C, 12 h)	169.7 mAh g^−1^ (1st discharge, 2.8–4.3 V, 0.1 C); 90% retention after 200 cycles (2.8–4.3 V, 0.5 C)	NA	[26b]
NCM111	Mechanochemically activation (boll milling, 500 rpm, 4 h); Sintering (800 °C, 10 h)	165 mAh g^−1^ (1st discharge, 2.5–4.3 V, 55.6 mA g^−1^); >80% retention after 100 cycles (2.5–4.3 V, 55.6 mA g^−1^)	NA	[56]
NCM111	Hydrothermal method (4 m LiOH solution, 220 °C, 4 h); Annealing (850 °C, 4 h)	≈155 mAh g^−1^ (1st discharge, 2.8–4.3 V, 155 mA g^−1^); 98% retention after 100 cycles (2.8–4.3 V, 51.6 mA g^−1^)	≈130 mAh g^−1^ (1st discharge, 2.8–4.3 V, 155 mA g^−1^); >94% retention after 100 cycles (2.8–4.3 V, 155 mA g^−1^)	[34c]
NCM111	Low‐temperature hydrothermal method (green reducing agents additives, 4 m LiOH solution, 100 °C, 4 h); Annealing (850 °C, 4 h)	157 mAh g^−1^ (1st discharge, 3–4.3 V, 0.1 C); 83% retention after 200 cycles (3–4.3 V, 1 C)	138 mAh g^−1^ (1st discharge, 2.8–4.2 V, 1 C); 87% retention after 100 cycles (2.8–4.2 V, 1 C);	[35]
NCM622		175 mAh g^−1^ (1st discharge, 3–4.3 V, 0.1 C); 92% retention after 60 cycles (3–4.3 V, 1/3 C);	154 mAh g^−1^ (1st discharge, 2.8–4.2 V, 1 C); 84% retention after 100 cycles (2.8–4.2 V, 1 C);	
NCM523	Hydrothermal (4 m LiOH solution, 220 °C, 3 h); Solid‐state eutectic molten‐salt solutions sintering (810 °C, 4 h)	150.5 mAh g^−1^ (1st discharge, 3–4.35 V, 150 mA g^−1^); 92% retention after 60 cycles (3–4.3 V, 50 mA g^−1^);	≥1.7 Ah, 190 Wh kg^−1^, 94.25% retention after 500 cycles (3–4.2 V, 150 mA g^−1^, pouch cell);	[57]
NCM523	Eutectic molten salts (LiOH/LiNO_3_, 300 °C, 4 h); Sintering (850 °C, 4 h)	149.3 mAh g^−1^ (1st discharge, 3–4.3 V, 150 mA g^−1^); 90.2% retention after 100 cycles (3–4.3 V, 150 mA g^−1^)	NA	[15]
NCM523	Molten salts (KCl/KNO_3_/LiNO_3_, 750 °C, 12 h)	161 mAh g^−1^ (1st discharge, 2.75–4.25 V, 30 mA g^−1^); 95.5% retention after 100 cycles (2.75–4.25 V, 30 mA g^−1^)	NA	[39]
NCM523	Eutectic molten salts (LiOH/Li_2_CO_3_, 440 °C, 4 h; 850 °C, 12 h)	146.3 mAh g^−1^ (1^st^ discharge, 2.8–4.3 V, 180 mA g^−1^); 89.06% retention after 200 cycles (2.8–4.3 V, 180 mA g^−1^)	NA	[55c]
NCM523	Eutectic LiI−LiOH salt, (Co_2_O_3_ and MnO_2_ additives, 200 °C, 4 h); Heat treat (850 °C, 5 h)	≈150 mAh g^−1^ (1st discharge, 2.5–4.3 V, 15 mA g^−1^); 80% retention after 200 cycles and 73% after 300 cycles (2.5–4.3 V, 75 mA g^−1^)	NA	[55a]
NCM523	Ternary hybrid molten salt (LiOH/LiNO_3_/CH_3_COOLi, 400 °C, 4 h); Annealing (850 °C, 5 h)	150 mAh g^−1^ (1st discharge, 3–4.3 V, 75 mA g^−1^); 93.7% retention after 100 cycles (3–4.3 V, 75 mA g^−1^)	NA	[55b]
NCM111	Ionothermal synthesis ([C_2_OHmim][NTf_2_]+LiBr, 150 °C); Calcination (500 °C, 4 h)	≈160 mAh g^−1^ (1st discharge, 3–4.3 V, 0.1 C);	≈139 mAh g^−1^ (1st discharge, 3–4.2 V, 0.1 C);	[17a]
NCM111	Redox mediate reaction (0.5 m DTBQ in DME, stirring for 60 min); Annealing (850 °C, 4 h)	171 mAh g^−1^ (1st discharge, 3–4.3 V, 16.1 mA g^−1^);	162.9 mAh g^−1^ (1st discharge, 1–2.8 V versus Li_4_Ti_5_O_12_, 16.1 mA g^−1^);	[46]

Fan et al. proposed a simple high‐temperature solid‐state sintering treatment with replenishing reagent LiOH to repair spent NCM523.^[^ ^24^ ^]^ The regenerated cathode delivered a 94.5% capacity retention after 100 cycles at a 1 C rate. Chi et al. developed a two‐step calcination process.^[^ ^26b^ ^]^ In the first stage of calcination, PVDF was fully removed; in the subsequent calcination step, the spent NCM111 cathode was recovered, with no additional compounds added to the repairing process. Meng et al. demonstrated an integrated method of mechanochemical activation and high‐temperature solid‐state sintering.^[^ ^56^ ^]^ Specifically, a ball‐milling process was introduced to facilitate the diffusion of lithium and nickel ions, thus decreasing the cationic disorder and rebuilding the layered crystal structure. Combined with sintering, the recycled spent NCM111 provided a specific discharge capacity of 165 mAh g^−1^ at 0.2 C at the first cycle.

**Figure 9:**
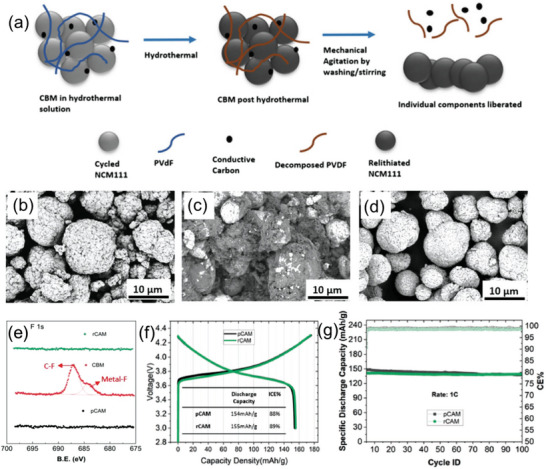
The integration of repair and pretreatment of spent NCM cathode. a) Illustration of the mechanism of decomposition of binder and cathode active material deliberation. SEM backscattering mode images of b) pCAM, c) CBM, and d) rCAM, respectively. e) F 1s XPS spectra of pCAM, CBM, and rCAM. The first cycle voltage profile f) of rCAM in half cell and cycling stability in full cell (g). Reproduced with permission.^[^ ^34c^ ^]^ Copyright 2022, Wiley‐VCH.

Compared with high‐temperature solid‐state sintering treatment, the hydrothermal relithiation method requires a much lower temperature. Pursuing lower energy consumption, researchers have highlighted this method. Gupta et al. proposed a hydrothermal‐annealing process to integrate the purification and direct repair of ternary cathode black mass (CBM).^[^ ^34c^ ^]^ The mature hydrothermal method was utilized not only for relithiation but also for degradation of PVDF, thus purifying the relithiated cathode particles, as shown in **Figure** **9**a. The backscattering‐mode SEM images of pristine cathode active material (pCAM), CBM, and recovered cathode active material (rCAM) are presented in Figures 9b–d, respectively. The SEM image of CBM demonstrated the coverage of PVDF on CAM. However, that of rCAM is similar to pCAM, demonstrating recovery of the particle. The XPS spectra indicated the effective removal of PVDF from rCAM (Figure 9e). In electrochemical tests, rCAM and pCAM showed similar specific capacities of 154 mAh g^−1^ at 1C in half cell test (Figure 9f). In full cell, rCAM exhibited a capacity retention of over 94% upon 100 cycles at 1 C (Figure 9g).

To reduce energy consumption, a low‐temperature hydrothermal relithiation at low pressure has been proposed by Yu et al.^[^ ^35^ ^]^ In this work, green additives, including ethanol, hydrogen peroxide, or ethylene glycol, were added to the hydrothermal systems to improve the redox kinetics, thus lowering the temperature and pressure, and improving the operational safety. The subsequent annealing treatment at 850 °C further improved the crystallinity of the ternary cathodes.

**Figure 10:**
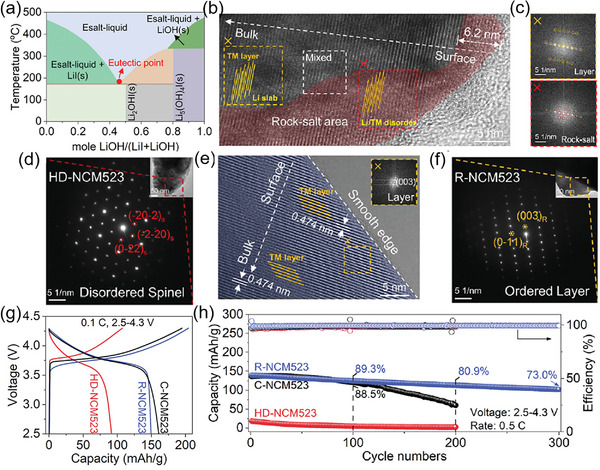
The repair of spent NCM523 via eutectic LiI‐LiOH salt. a) The phase diagram of the LiI‐LiOH system. b) HRTEM image of HD‐NCM523. c) The FFT patterns from corresponding areas. d) SAED pattern of HD‐NCM523. e) HRTEM pattern and f) SAED image of R‐NCM523. g) The first voltage profiles and h) long‐term stability of HD‐NCM523, R‐NCM523, and C‐NCM523, respectively. Reproduced with permission.^[^ ^55a^ ^]^ Copyright 2022, American Chemical Society.

The newly developed molten salt medium thermochemical method has been announced for relithiating and direct repairing ternary cathode.^[^ ^39^ ^]^ This method aimed at directly relithiating spent NCM523 and removing carbon impurities. Nevertheless, the operating temperature is relatively high up to 750 °C, which is close to that of the high‐temperature solid‐state sintering method. The eutectic salt mixture has been proposed to lower the operating temperature of the direct repairing cathode.^[^ ^55a^ ^]^ The setting of the molten salt ratio is guided by a phase diagram, as shown in **Figure** **10**a, from which it can be seen that the eutectic salt mixture has the lowest melting point. It provides a homogeneous liquid environment, thus accelerating the ion diffusion and prompting the thermochemical reaction at a relatively low temperature, which reduces energy consumption. Ma et al. proposed a method of combining the eutectic LiI‐LiOH salt and additives (Co_2_O_3_ and MnO_2_) to directly repair NCM523 at 200 °C.^[^ ^55a^ ^]^ The microstructural characterization of highly degraded NCM523 (HD‐NCM523) and repaired NCM523 (R‐NCM523) demonstrated the efficiency of this method. The disordered spinel and rock‐salt structures were presented in the HRTEM image of HD‐NCM523 (Figure 10b), and confirmed by fast Fourier transform (FFT) patterns (Figure 10c) and selected area electron diffraction (SAED) patterns (Figure 10d). In contrast, the R‐NCM523 had an ordered layered phase (Figure 10e,f), indicating the recovery of lattice through this eutectic salt thermochemical method. R‐NCM exhibited a comparable discharge capacity to commercial NCM523 (C‐NCM523) at the first cycle (Figure 10g). Surprisingly, it provided a better long‐term cycling performance than C‐NCM523, delivering a 73% capacity retention after 300 cycles at 0.5 C (Figure 10h).

Based on the molten salt thermochemical method, other innovative processes for repairing NCM cathodes have been proposed. A ternary molten salt medium^[^ ^55b^ ^]^ was introduced in the repairing system to lower the operating temperature. A combination of hydrothermal relithiation and eutectic molten salt sintering was proven effective for repairing spent NCM ternary cathodes.^[^ ^57^ ^]^

Another innovative repairing method combines ionothermal relithiation without further calcination,^[^ ^17a^ ^]^ of which the used IL could be recycled and reused. The operating temperature is 150 °C during the ionothermal relithiation which is quite energy‐saving. Subsequent filtering and washing processes are required to remove organic solvents.

### 3.3 Direct Repair of LCO and LMO

LCO and LMO are important cathode materials mainly applied in portable electronics. Representative publications for directly repairing LCO are listed in **Table** **2**, covering high‐temperature solid‐state calcination, electrochemical relithiation, hydrothermal method, chemical relithiation, and DES method.

**Table 2:** Representative works for directly repairing spent LCO.

Direct repairing method	Half‐cell electrochemical performance	Full‐cell electrochemical performance	Ref.
Electrochemical relithiation (Li_2_SO_4_ solution); Calcination (700 °C, 2 h)	136 mAh g^−1^ (1st discharge, 3–4.3 V, 0.2 C); ≈98% retention after 100 cycles (3–4.3 V, 0.2 C)	NA	[43]
Electrochemical relithiation (Li_2_SO_4_, LiOH solution); Calcination (700 °C, 6 h)	140 mAh g^−1^ (1st discharge, 3–4.2 V, 0.1 C); 93% retention after 100 cycles (3–4.2 V, 0.1 C)	NA	[58]
Hydrothermal method (4 m LiOH Solution, 220 °C, 4 h); Annealing (800 °C, 4 h)	153.1 mAh g^−1^ (1st discharge, 3–4.3 V, 15 mA g^−1^); 91.2% retention after 100 cycles (3–4.3 V, 150 mA g^−1^)	NA	[34a]
Deep eutectic solvent (LiCl–CH_4_N_2_O, 120 °C, 4 h); Annealing (850 °C, 2 h)	133.1 mAh g^−1^ (1st discharge, 2–4.2 V, 14 mA g^−1^); 72.7% retention after 100 cycles (2–4.2 V,70 mA g^−1^)	NA	[17c]
Auto‐oxidative relithiation (DMSO‐LiBr, 150 °C, 8 h); Calcination (150 °C, 12 h)	155.384 mAh g^−1^ (1st discharge, 3–4.3 V, 0.1 C); 90.79 retention after 100 cycles (3–4.3 V, 1 C)	NA	[45]
High‐temperature solid state calcination (Li_2_CO_3_ addictive, 900 °C, 6 h)	140 mAh g^−1^ (1st discharge, 3–4.2 V, 14 mA g^−1^); >80% retention after 100 cycles (3–4.2 V, 70 mA g^−1^)	223.1 mAh, 190 Wh kg^−1^, ≈67% retention after 100 cycles (2.75–4.2 V, 28 mA g^−1^, pouch cell);	[25]
Two‐stage molten salt heating (300 °C for 8 h; 500 °C for 16 h)	144.5 mAh g^−1^ (1st discharge, 2.75–4.25 V, 30 mA g^−1^); 92.5% retention after 100 cycles (2.75–4.25 V, 30 mA g^−1^)	NA	[26c]
Ternary molten salt (LiOH–KOH–Li_2_CO_3_, 500 °C, 8 h);	149.1 mAh g^−1^ (1st discharge, 2.75–4.25 V, 30 mA g^−1^); 93% retention after 200 cycles (2.75–4.25 V, 30 mA g^−1^)	NA	[59]
Ball‐milling spray; Heat treatment (800 °C, 4 h)	165 mAh g^−1^ (1st discharge, 2.8–4.5 V, 0.2 C); 72.7% retention after 100 cycles (3–4.3 V, 0.2 C)	NA	[60]

Recently, Wang et al. proposed a closed recycling loop for both regenerations of anode and cathode materials.^[^ ^25^ ^]^ The high‐temperature solid‐state method was utilized for repairing spent LCO. The difference with the conventional high‐temperature solid‐state method lies in the use of an extra Li source, which was converted from the retired batteries. The repaired LCO cathode exhibited a comparable capacity to commercial LCO cathode in pouch cell test. In addition, closed‐loop cycling provides the potential for a low‐cost and green generation method. Similar to the direct repair process for LFP and ternary cathodes, high‐temperature solid‐state calcination has been an important procedure in the direct repair of retired LCO. The calcination often works as a post‐procedure of other relithiation processes including electrochemical relithiation,^[^ ^43^, ^58^ ^]^ hydrothermal relithiation,^[^ ^34a^ ^]^ chemical relithiation,^[^ ^45^ ^]^ and DES method.^[^ ^17c^ ^]^

Electrochemical relithiation has been utilized for direct repairing LCO. Yang et al. confirmed that the electrochemical relithiation process was controlled by a charge transfer process or a diffusion process when the electrolyte solution concentration was high or low, respectively.^[^ ^43^ ^]^ The crystal structure of relithiated LCO was fully recovered by a subsequent annealing treatment, exhibiting a capacity of 136 mAh g^−1^ at 0.2 C, which is comparable to that of a commercial LCO cathode. In this work, the asymmetric relithiation system was fabricated, where the spent LCO cathode and Pt plate were working and counter electrodes, respectively. During the LCO electrochemical relithiation, the counter electrode underwent an oxygen evolution, which decreased the PH of the aqueous electrolyte, thus eroding the Al current collectors. In this regard, a symmetric electrochemical relithiation system was developed by Yang et al, where the spent LCO cathode worked as both the working electrode and counter electrode.^[^ ^58^ ^]^ This design avoided the generation of oxygen and no bubbles were seen during the electrochemical relithiation process.

Fei et al. proposed a chemical relithiation, specifically speaking, an auto‐oxidation strategy for direct regenerating spent LCO, utilizing DMSO and LiBr. LiBr provided extra Li^+^ and DMSO acted as solvent and oxygen donor.^[^ ^45^ ^]^ The auto‐oxidative DMSO with nucleophilic properties can accelerate the release of Li^+^ in LiBr. Additionally, DMSO with its cosolvent property provided a high charge flux medium to transport the Li^+^ and O^2−^ to spent cathode materials. It is worth mentioning that the operating temperature is as low as that of the hydrothermal method (150 °C).

**Figure 11:**
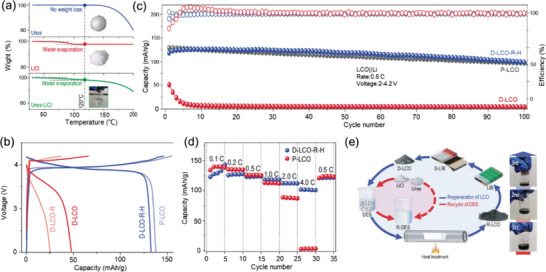
The repair of spent LCO via DES. a) TG curves of CH_4_N_2_O, LiCl, and DES. b) Charge–discharge profiles, c) long‐term cycling performances, and rate performances of several types of LCO, in which D‐LCO‐R‐H and p‐LCO represent the repaired and heated LCO and pristine LCO, respectively. e) Recycling and reuse procedure of DES. Reproduced with permission.^[^ ^17c^ ^]^ Copyright 2022, Oxford Academic.

The newly developed eutectic molten salt with a low melting point has been proposed to lower the operating temperature for repairing degraded cathodes. Wang et al. proposed a recyclable LiCl‐CH_4_N_2_O DES to directly repair the degraded LCO cathode.^[^ ^17c^ ^]^ The TG curves (**Figure** **11**a) showed that almost no weight loss in the DES was observed below the temperature of 150 °C, guiding the temperature setting of the repair procedure. The repaired LCO delivered an initial discharge capacity of 133.1 mAh g^−1^ at 0.1 C, which was close to that of pristine LCO (Figure 11b). Meanwhile, the long‐term stability of repaired LCO was comparable to pristine LCO, exhibiting a capacity retention rate of 90% after 100 cycles at 0.5 C (Figure 11c). It is worth noting that the repaired LCO exhibited a better rate performance than the pristine LCO (Figure 11d). Moreover, the DES could be recycled and reused, as shown in Figure 11e. Overall, DES provides new insight into direct cathode repair in a more environmentally friendly and cost‐effective way.

Only a few works have been so far published on the direct repair of spent LMO cathode. Wang et al. performed a comparative study of repairing aged LMO cathodes using high‐temperature solid‐state and the hydrothermal method, suggesting the superiority of the hydrothermal method.^[^ ^61^ ^]^ Gao et al. proposed a one‐step hydrothermal regeneration using a diluted Li‐containing solution,^[^ ^34d^ ^]^ which enabled the reconstruction of desired stoichiometry and microphase purity. More works are needed in this aspect.

## 4 Upcycling Strategies

Apart from the direct regeneration of spent cathodes to their pristine states, the upcycling of spent cathodes is also critical to reaching the sustainability of LIBs. On the one hand, upcycling retired cathodes to obtain an improved electrochemical performance meets the increasing demands of practical applications. For example, the EVs require higher and higher energy density LIBs for longer mileage, thus the ternary oxide cathodes have developed from first‐generation, outdated NCM111 to Ni‐rich NCM. On the other hand, due to the intrinsic merits of the spent cathode, including composition, crystalline structures, and defects, they can be applied to other fields after certain modifications.

### 4.1 Improving Performance

For the LCO cathode, the mainstream upcycling target is to increase the electrochemical stability under high voltage. The aims of upcycling the ternary cathode are increasing the Ni content and transforming it into single‐crystal particles, and for LMO, the upcycling work is mainly focused on the suppression of the Jahn–Teller effect of Mn. LFP is one of the most widely used LIBs cathode materials. The spent LFP shall be in a huge mass in the coming years considering its dominant application in electric vehicles. As for LFP, although there are few related papers, some patents have disclosed the strategy of Li‐supplementary and Mn‐doping simultaneously, resulting in Mn‐doped LFP with the merit of higher working voltage. Such is worthy of academic attention.

#### 4.1.1 Toward High‐Voltage LCO

**Figure 12:**
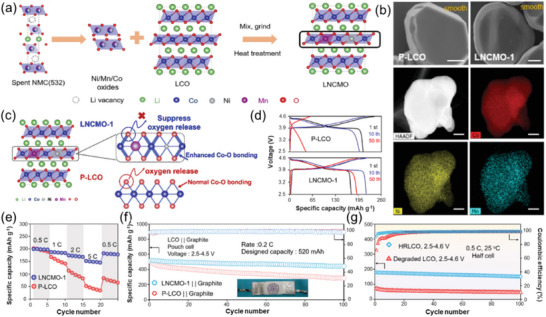
High voltage LCO from degraded materials. a) Schematic of the fabrication process for high‐voltage LCO cathode materials; b) SEM images of typical particles of P‐LCO and LNCMO, elemental mapping of an LNCMO particle; c) Schematic of the strengthened Co─O bonding and thus suppressed oxygen release of cathode materials after the introduction of substitutional Ni/Mn dopants in the Co layer; d) Voltage profiles of P‐LCO (top) and LNCMO‐1 (bottom) at 0.5 C from 2.5 to 4.6 V; e) Rate performances and long‐term cycling performances in full‐cell f) of P‐LCO and LNCMO‐1, respectively. g) Cycling performance of HRLCO in half‐cell. Reproduced with permission.^[^ ^27^ ^]^ Copyright 2022, American Chemical Society.

LCO suffers from poor recycling stability under high voltage, which hinders its further development in high energy.^[^ ^62^ ^]^ The shortcoming is intrinsically derived from the insufficient Co─O bonding at high voltage,^[^ ^63^ ^]^ leading to the lattice oxygen release^[^ ^64^ ^]^ as well as lattice distortion.^[^ ^65^ ^]^ In that case, many methods have been proposed to overcome the obstacle, among which element doping proves to be efficient.^[^ ^62^, ^63^, ^66^ ^]^ Inspired by this idea, Jia et al. fabricated a Ni/Mn doped high‐voltage LCO (LNCMO‐1, 1 refer to the 1% doping ratio) capable of cycling stably under 4.6 V, where the doping elements were originated from spent NCM523 cathode,^[^ ^27^ ^]^ as shown in **Figure** **12**a. The SEM images and EDX element mapping of pristine LCO (P‐LCO) and LNCMO‐1 prove that Ni and Mn were evenly doped into P‐LCO (Figure 12b). The improved stability under high voltage is attributed to the enhanced Co─O bonding, thus suppressing the lattice oxygen release (Figure 12c). The voltage profiles of P‐LCO (top) and LNCMO‐1 (bottom) at 0.5 C from 2.5 to 4.6 V demonstrated improved electrochemical stability under high voltage conditions (Figure 12d). In addition, LNCMO‐1 exhibited superior rate performance compared to P‐LCO (Figure 12e). The long‐term cycling performance of LNCMO‐1 in pouch‐cell testing was also superior to that of P‐LCO (Figure 12f). It is worth noting that this strategy was successfully applied to upgrade the spent LCO cathode, demonstrating the superior long‐term cycling performance under high‐voltage (Figure 12g). The doping strategy has also been prove valid to improve the electrochemical performance of LCO in other studies.^[^ ^29^, ^36^, ^67^ ^]^

Targeting the high‐voltage issue, interface engineering has also been proposed to achieve high‐voltage stability of LCO.^[^ ^68^ ^]^ Wang et al. proposed an adaptive hydrothermal relithiation method combined with short annealing post‐treatment to regenerate spent LCO with mixed phases (Co_3_O_4_/Co_2_AlO_4_, CoTiO_3,_ and Li_3_PO_4_) coating on the surface.^[^ ^31^ ^]^ Specifically, the coating precursors Li_1.4_Al_0.4_Ti_1.6_(PO_4_)_3_ were mixed with relithiated LCO and then annealed. The final regenerated LCO was coated by mixed phases and exhibited an initial discharging capacity of 166 mAh g^−1^ and a discharging capacity retention of 93% at 1 C (1 C = 150 mA g^−1^) for 100 cycles with a voltage range of 3–4.4 V. Similarly, a nano Al_2_O_3_‐coated LCO was regenerated from spent LCO electrode, of which the electrochemical performance was enhanced.^[^ ^30^ ^]^ Furthermore, Fei et al. proposed a novel synchronous modification to directly regenerate spent LCO materials, which was doped by Mg/Ti and coated by a cladding layer composed of Al_2_O_3_ and LiCo_1−_ *_y_* Al*_y_* O*_z_* solid solution.^[^ ^69^ ^]^ The regenerated LCO cathode exhibited excellent electrochemical stability due to the inhabitation of the interface side reaction at a high cutoff voltage of up to 4.5 V.

In short conclusion, targeting the instability issue at high cutoff voltage, two catalogs of methods (doping and surface engineering) have been proposed to direct upcycle the spent LCO cathode. On the one hand, these methods are mature in the existing production line, exhibiting scalability. On the other hand, spent LCO cathodes were reborn with superior electrochemical performance, fitting with the goal of carbon neutrality and sustainability.

#### 4.1.2 Toward Ni‐Rich and Single‐Crystal Ternary Cathode

**Figure 13:**
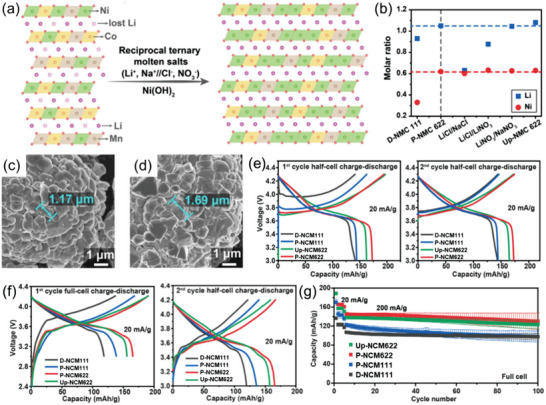
Flux upcycling of spent NCM111 to nickel‐rich NCM cathodes in reciprocal ternary molten salts. a) Illustration of the upcycling of D‐NCM111 to Up‐NCM622 in RTMS. b) Metal molar ratios of D‐NCM111 and P‐NCM622 products obtained from different binary molten salts, and Up‐NCM622 based on ICP results. c,d) SEM images of D‐NCM111 and Up‐NCM622. e) The first cycle charge/discharge curves of NCMs for half‐cell tests. f,g) The first cycle charge/discharge curves and cycle performance of NCMs for half‐cell and full‐cell tests. Reproduced with permission.^[^ ^18a^ ^]^ Copyright 2022, Elsevier.

A general developing trend of ternary cathodes is from Ni‐contained to Ni‐rich cathodes since NCM111 and LMO are gradually obsolete for new applications.^[^ ^70^ ^]^ With the retire of EOL EV batteries, a significant supply of spent NCM111 will occur. With the aim of direct upcycling spent NCM111 to Ni‐rich NCM cathodes, Dai's group developed a reciprocal ternary molten salts (RTMS) system to simultaneously realize the addition of Ni and relithiation of the spent NCM111.^[^ ^18a^ ^]^ Different from the direct recycling process where only a slight weight change occurred, the upcycling of NCM111 to Ni‐rich NCM is accompanied by a significant weight change and reconstruction process. The challenge is even tougher as an oxidation environment is needed to transfer Ni^2+^ to Ni^3+^ to minimize lattice oxygen vacancies and Li/Ni anti‐site defects in Ni‐rich NCM. Fortunately, the applied Li^+^, Na^+^//Cl^−^, NO_3_^−^ system can work in a wide temperature range, offer sufficient Li source, and provide an oxygen‐rich environment, and therefore successfully upcycled the spent NCM111 to Ni‐rich NCM (**Figure** **13**a). The Li:Ni molar ratio of delithiated NCM (D‐NCM), products synthesized in diverse systems, and upcycled NCM (Up‐NCM) was detected by ICP (Figure 13b), demonstrating that the combined Li^+^, Na^+^, Cl^−^, and NO_3_^−^ is critical for the successful upcycling of D‐NCM111 to NCM622 and NCM811. The SEM observation revealed that the primary particles of Up‐NCM622 become larger than those of D‐NCM111 (Figure 13c,d), suggesting the growth of primary particles during the upcycling process. In half‐cell tests, the initial capacity of D‐NCM111 and pristine NCM (P‐NCM)111 is inferior to Up‐NCM622 and commercial P‐NCM622 using 20 mA g^−1^ current density and a voltage range of 3–4.3 V. Similar results were obtained for the following cycles (Figure 13e), and the Up‐NCM622 showed the best cycling performance among the studied samples, confirming the sustainable superior performance of Up‐NCM622 in half‐cell tests. In further full‐cell experiments, Up‐NCM622 exhibited higher capacities than both P‐NCM111 and D‐NCM111 under either 20 or 200 mA g^−1^, and its performance is comparable to P‐NCM622 (Figure 13f,g).

**Figure 14:**
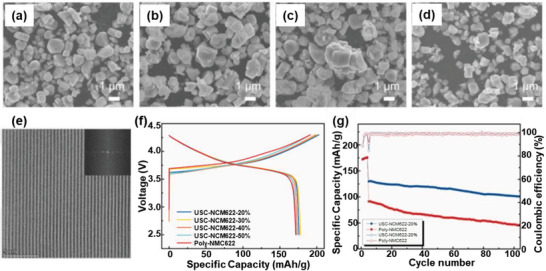
Direct upcycling of mixed Ni‐lean polycrystals to single‐crystal Ni‐rich cathode materials. a–d) The morphology of USC‐NCM622 with 20%, 30%, 40%, 50% excess LiOH. e) HAADF‐STEM image of USC‐NCM622‐20% and the inset image of the FFT pattern of USC‐NCM622‐20%. f) Voltage profiles at the first cycle of pristine sample and with different amounts of excess LiOH. g) Cycling performance of USC‐NCM622‐20% and P‐NCM622 at the current rate of 5 C. Reproduced with permission.^[^ ^71^ ^]^ Copyright 2022, Elsevier.

The above study sets a benchmark since it opens a new era of recycling LIBs by compositional adjustment. Using an RTMS system, the spent cathode has almost been reconstructed, while maintaining the initial layered framework. A remaining challenge is that the collected spent NCM cathodes are often mixed compounds with many phases, of which the shredding and sorting process cannot separate each component.^[^ ^7^ ^]^ Therefore, a generalizable method is urgently needed to upcycle discarded ternary cathodes. In this aspect, Wang's group reported a one‐step molten salt method that successfully converted spent mixed polycrystalline Ni‐lean cathodes into single‐crystal Ni‐rich cathodes without sorting processes.^[^ ^71^ ^]^ By varying the composition of the molten salt, upcycled single crystal NCM622 in different sizes have been obtained (**Figure** **14**a–d). A typical HAADF‐STEM image showed that the obtained sample displayed an ideal layered structure with good crystallinity (Figure 14e). All samples exhibited similar charge–discharge profiles to the commercial polycrystalline NCM622 (P‐NCM622) with no obvious plateau or capacity difference (Figure 14f). Moreover, the best sample (USC‐NCM622‐20%) demonstrated much better cycling performance than P‐NCM622 (Figure 14g).

#### 4.1.3 Suppressing Jahn–Teller Effect of Mn in LMO

**Figure 15:**
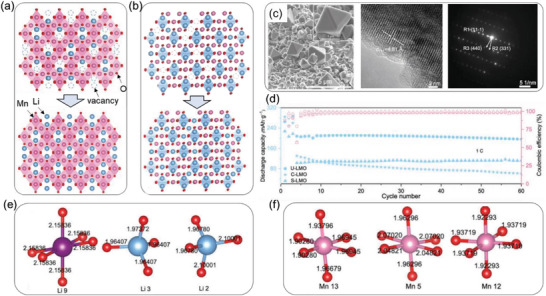
Stabilization spent cathode materials by In Situ Li/Mn Disorder. Illustration of a) the hydrothermal lithiation process in which Li^+^ is re‐dosed to the Li‐deficient sites to recover its desired stoichiometry, and b) the upcycling process in which Li^+^ is re‐invested into the Mn‐deficient site to substantially enhance its electrochemical properties. c) SEM images, an HR‐TEM image, and a SAED image of upcycled LMO materials. The states of e) Li tetrahedra and f) Mn octahedra in disordered LMO materials. Atomic bond lengths are in Å. Reproduced with permission.^[^ ^73^ ^]^ Copyright 2022, Wiley‐VCH.

As the cheapest oxide cathode material for LIBs, spinel LMO has been widely applied in electric bicycles where the low cost has compensated for its performance inferiority compared with layered oxides.^[^ ^72^ ^]^ The spent LMO (S‐LMO) is identified to have substantial Mn deficiency and cationic disorder. Traditional repair by high‐temperature solid‐state reaction or hydrothermal method simply redoes Li^+^ to Li‐deficient sites to recover its original stoichiometry (**Figure** **15**a). Li et al. innovated recycling by utilizing a low‐carbon‐economy technique to insert Li^+^ into both Li^+^‐vacancy and Mn^2+^‐vacancy (Figure 15b).^[^ ^73^ ^]^ The resulting upcycled samples (U‐LMO) were evenly distributed LMO single particles with rectangular shapes, and their lattice and phase were confirmed by HRTEM and SAED (Figure 15c). Impressively, the U‐LMO displayed much better electrochemical performance than both S‐LMO and commercial LMO (C‐LMO). It delivered a discharge capacity of 212 mAh g^−1^ after 100 weeks of cycling at 2 C. Theoretical simulation on the geometric structure and corresponding electronic structure of U‐LMO revealed that the improved performance originated from the electron rearrangement around the atoms caused by Li/Mn disorder. Three different chemical environments of Li and Mn were identified in the disordered LMO structure (Figure 15e,f). Such change also leads to easier Li‐migration and the suppressed Jahn–Teller distortion of Mn, thus improving the cycling stability.

### 4.2 New Applications

#### 4.2.1 Alkali‐Ion Storage

**Figure 16:**
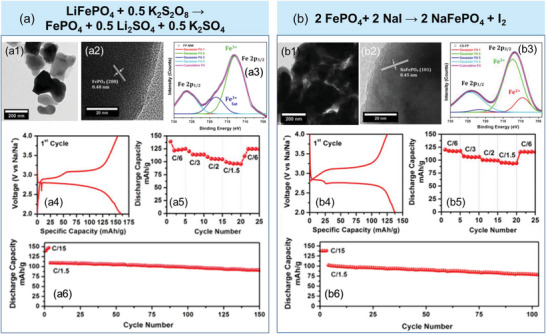
Reuse of LFP cathode from spent LIBs for rechargeable SIBs. a) Obtaining FePO_4_ by a microwave method. b) Preparing NaFePO_4_ from chemically sodiated FePO_4_. a1,b1) Low‐magnification TEM images, a2,b2) high‐resolution TEM images, a3,b3) high‐resolution Fe 2p XPS spectra, a4,b4) 1st cycle charge–discharge profile at C/15, a5,b5) rate performance, and a6,b6) cycling performance. Reproduced with permission.^[^ ^74^ ^]^ Copyright 2021, American Chemical Society.

LFP cathode has been recycled to store other alkali‐ion such as Na^+^. For example, the LFP cathode from spent LIBs was treated by a low‐temperature, rapid (15 min) microwave process.^[^ ^74^ ^]^ TEM and XPS characterization demonstrated the formation of fully delithiated phase FePO_4_ (**Figure** **16**a1–3). The FePO_4_/Na half‐cell displayed a charge plateau at 3.1 V and a discharge plateau at 2.7 V with a discharge capacity close to the theoretical value (159 mAh g^−1^) (Figure 16a4), good rate performance (Figure 16a5), and retained 85% of its initial capacity even at the end of the 150th cycle (Figure 16a6). This is comparable with that of the fresh NaFePO_4_ (NFP) electrodes reported in the literature. Further, to compensate for the losing Na in the microwave‐derived FePO_4_, facile chemical sodiation was performed using NaI as the reducing agent and sodium source, as formulated in Figure 16b. The product was checked by TEM and XPS (Figure 16b1–3), proving the success of the sodiation treatment. In the electrochemical test, it displayed similar voltage profiles to fresh NFP, with a slightly lower specific capacity (125 mAh g^−1^) (Figure 16b4). Meanwhile, the rate and cycling performance (Figure 16b5,6) are consistent with the electrochemically sodiated sample. It illustrates that a commercially viable chemical sodiation process is adoptable to recycle materials from spent LIBs.

#### 4.2.2 Catalysts for OER

**Figure 17:**
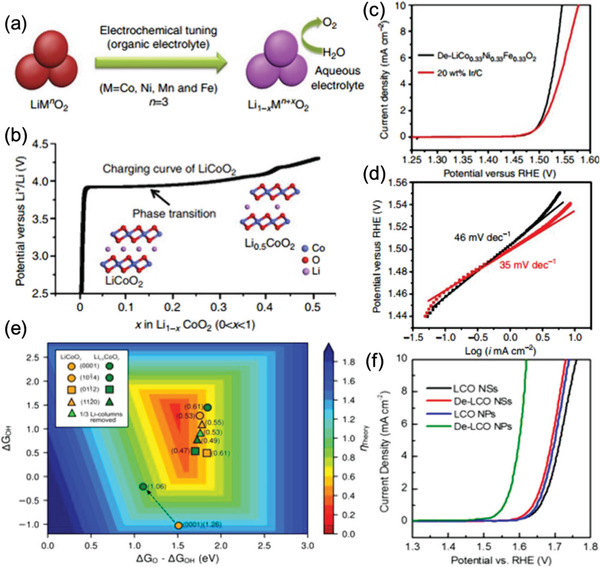
Electrochemical tuning of layered lithium transition metal oxides for improvement of OER. a) Schematic illustration of the electrochemical tuning process of LiMO_2_ (M = Co, Ni, Mn, and Fe), where the oxidation state of M center would be higher after electrochemical tuning in organic electrolyte and suitable for water oxidation in aqueous electrolyte. b) A typical charging curve of LCO. c) Comparison of polarization curves of delithiated LiCo_0.33_Ni_0.33_Fe_0.33_O_2_ and 20 wt% Ir/C catalysts. d) Tafel slopes of De‐LiCo_0.33_Ni_0.33_Fe_0.33_O_2_ and 20 wt% Ir/C catalysts. e) Calculated OER activity map of the LCO/Li_0.5_CoO_2_ system. The orange (green) symbols position the activity of the individual surface of LiCoO_2_ (Li_0.5_CoO_2_) with the value of theoretical OER overpotential (*η*_Theory_) shown in brackets. The arrows indicate the change in the activity upon delithiation. f) Polarization curves of LCO NSs and NPs before and after delithiation, respectively. (a–d): Reproduced with permission.^[^ ^75^ ^]^ Copyright 2014, Springer Nature. (e,f): Reproduced with permission.^[^ ^76^ ^]^ Copyright 2017, American Chemical Society.

Co‐based compounds have been proven to be competitive catalysts to precious metal oxides as a result of their good oxygen evolution reaction (OER) activity, easy accessibility, and abundance. Previous studies have shown that the OER activity is highly related to the electronic structure of the Co atom, including oxidation state, for example, filling and O p‐band center. As spent LCO or ternary cathodes are usually characterized by the loss of Li and defects, they may serve as good OER catalysts. Although there are no direct investigations, Cui et al. reported the OER process using the electrochemically delithiated LCO and ternary oxides (**Figure** **17**a), which can mimic the spent LIBs oxide cathodes in many aspects.^[^ ^75^ ^]^ Taking LCO as an example, approximately half of the Li in the LCO was extracted, leading to the formation of Li_1−_ *_x_* CoO_2_ (*x* = 0.5) (Figure 17b). The crystal structure of the material and the electronic structure of the Co atom were both changed after phase transition, which occurred at the potential of 3.9–4 V versus Li^+^/Li. In OER tests, it showed impressive activity and good durability. The most efficient OER catalyst identified was based on delithiated LiCo_0.33_Ni_0.33_Fe_0.33_O_2_, which possessed a small onset potential of 1.47 V versus reversible hydrogen electrode (RHE) and a low Tafel slope of 35 mV dec^−1^. After delithiation, the LCO nanoparticles (NPs) show a noticeably enhanced OER activity, while the LCO nanosheets (NSs) exhibit negligible OER enhancement, demonstrating that the basal plane of delithiated LCO is inactive while other major surfaces are active for OER. Impressively, a commercial 20 wt% Ir/C catalyst with the same mass loading exhibited a similar onset potential, but a larger Tafel slope (Figure 17c,d). Therefore, the prepared Li‐deficient oxide cathode for LIBs is even superior to the Ir/C catalyst to drive significant anodic current densities for OER. Later, the enhanced OER performance was investigated by theoretical simulations, revealing the active sites for catalytic processes (Figure 17e), and confirmed by experiments on morphology‐controlled LCO (Figure 17f).^[^ ^76^ ^]^

#### 4.2.3 Catalysts for Zn–Air Batteries

**Figure 18:**
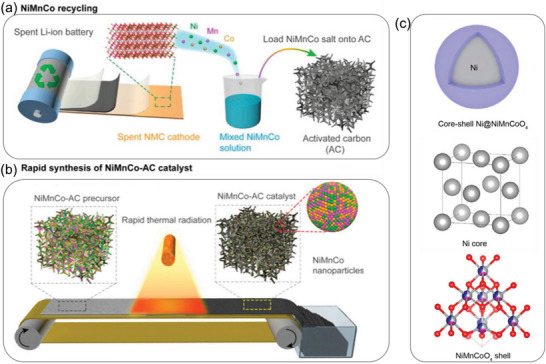
Recycling spent LiNi_1−_ *_x_*_−_ *_y_* Mn*_x_* Co*_y_* O_2_ cathodes to bifunctional NiMnCo catalysts for zinc–air batteries. a) Recovery of the NiMnCo from spent LIBs. A mixed NiMnCo solution was obtained by dissolving a spent NCM532 cathode in nitric acid. Then, the NiMnCo salt was loaded on AC to prepare a NiMnCo‐AC precursor. b) Schematic of the rapid thermal radiation method to synthesize the NiMnCo‐AC catalyst continuously. The NiMnCo‐AC precursor was driven by a conveyor and moved through a radiative heating zone. After that, NiMnCo nanoparticles were formed and uniformly dispersed on the surface of the AC support. c) The identified core–shell structure of the NiMnCo nanoparticle. Reproduced with permission.^[^ ^18b^ ^]^ Copyright 2022, Proceedings of the National Academy of Sciences.

Zhou's group has employed a rapid thermal radiation method to convert the spent LIBs into highly efficient bifunctional NiMnCo‐activated carbon as a catalyst for zinc–air batteries.^[^ ^18b^ ^]^ The spent NCM cathodes were first collected from spent LIBs, dissolved into nitric acid to obtain a mixed NiMnCo solution, and then loaded on active carbon (AC) support (**Figure** **18**a). Afterward, it was transferred to a carbon cloth and moved quickly across a high‐temperature radiative heating zone to form the catalyst (Figure 18b). It exhibited a unique core–shell structure where the core is made of face‐centered cubic Ni while the shell is made of spinel NiMnCoO_4_ (Figure 18c). The core–shell structure redistributes the electronic structure of the shell, thus decreasing the energy barrier for oxygen reduction reaction (ORR) and OER, and therefore showed excellent electrochemical performance in zinc–air batteries. In a flexible zinc–air battery, a high powder density of 187.7 mW cm^−2^, a low voltage gap of 0.72 V, and a long duration of 200 h have been achieved. This work demonstrates a prototype reuse of spent ternary cathodes as catalysts for energy conversion devices. It can be expected that other metal–air or metal–O_2_ batteries will be developed mimicking this strategy.

#### 4.2.4 Other Application

In addition to the upcycling mentioned above, spent LIBs cathodes have also been developed for other applications. A representative example is using spent LCO as a solid lubricant additive. After mixing thoroughly with graphene and Aremco binder in an excess volatile organic solvent, Parikh et al. found that the new composite solid lubricant exhibited exceptional lubricious properties in tribology.^[^ ^77^ ^]^ Another interesting study is using spent LFP or LMO in water purification technologies.^[^ ^78^ ^]^ In addition, a novel LFP‐graphite, which integrated the renewable cathode LFP and anode graphite from the spent LFP battery into the cathode material of the new dual ion battery (DIB), has been reported recently.^[^ ^79^ ^]^ Thanks to the porous structure and the vacant interstitial sites, the spent LIB cathodes offer sufficient adsorption sites for heavy metal adsorption, while requiring little chemical pretreatment, activation, or hazardous materials. Such spent‐batteries‐based adsorbents could be effective for a broad range of water pollution such as heavy metals, pesticides, and fertilizers. From another view, Liu et al. applied a delithiated LFP for Li^+^ extraction from seawater, which offers a fresh and promising recycling strategy.^[^ ^80^ ^]^

## 5 Perspective

Recycling spent LIBs cathode materials have been progressed forward to a large step during the last few years. By repairing or upcycling the EOL cathode materials, the valuable components can be returned to the market more efficiently compared to traditional pyrometallurgy or hydrometallurgy, which means being quicker and with relatively lower extra cost. Despite these advances, there remain some issues to be addressed to achieve the long‐term sustainability of the LIBs industry.

1) Repair of the spent LIBs cathode suits best to those maintaining structural completeness, where inside defects or surface impurities are allowed. Whereas, the feasibility of upcycling depends on the specific application field. Especially for the wide‐used high‐temperature solid‐state method, accurate composition information is essential for repair. Therefore, a convenient, non‐destructive, and quick detection method is needed to differ the defects‐included cathode from the seriously damaged ones and to expose precise composition information so that a suitable recycling method can be applied accordingly.
2) In retrospect of the history of the LIBs industry, the EOL LIBs will be more diverse, containing LCO, LFP, NCM111, NCM532, NCM622, LiNi_*x*_Co_*y*_Al_1−*x*−*y*_ (NCA), etc. The current pre‐treatment of the EOL LIBs needs little differential processing for pyrometallurgic or hydrometallurgical recycling, so the recollected LIBs can be mixed and go through a uniform pre‐treatment. In contrast, the so‐far‐developed repairing or upcycling methods are only demonstrated on some specific cathodes. Therefore, the recollected EOL LIBs need to be classified before being disassembled. Otherwise, more generalizable repairing/upcycling methods are needed for mixed spent LIBs cathode.
3) The work‐flow of the repairing or upcycling procedures is waiting for further simplification to improve the efficiency. As is well known, the EOL LIBs cathode (black mass) generally contains many impurities such as conductive carbon, binder, residual electrolyte, and cathode‐electrolyte interface. A purification process, such as heating or solvent washing, is needed before traditional recycling, but often accompanied by side effects. What is worse, the exploited repairing or upcycling method itself can hardly be considered sustainable. To tackle this issue, on the one hand, an integrated method has been reported that merges the purification and relithiation processes.^[^ ^34c^ ^]^ On the other hand, effective separation of cathode active materials benefits the impurities control during repairing and upcycling procedures.^[^ ^81^ ^]^ Inspired by this, more innovative and simplified strategies shall be developed in the future.
4) The methods of cathode direct regeneration are supposed to be improved toward low energy consuming and low CO_2_ emission. For instance, lowering the reacting temperature and pressure by hydrothermal method with the premise of the relithiation effectiveness will consume less energy, emit less CO_2_, and thus improve the economics of the industry. As for molten salt thermodynamics, how to recycle the post‐Li‐containing salts becomes urgent, which is expensive and costing. DES becomes a potential choice, as the recycling of used DES has been reported.
5) The intrinsic mechanisms of direct regeneration of spent cathodes are worth exploring. Current works focus on the electrochemical improvement of the cycled cathodes, however, the mechanism of the cathode recovering is seldom elucidated. Combining in situ or operando techniques, we hope that the intrinsic mechanism in this area will be disclosed, thus directing the future recycling of spent LIBs.
6) Whether different cathode materials have their own most suitable recycling methods is still a pending question. The reason lies in insufficient investigation of repairing mechanisms, lack of unified evaluation criteria, and little consideration of economic effectiveness. More studies are needed to build a close relationship in between.
7) There are little demonstration of the scaling‐up verification of the proposed repairing or upcycling methods. As lab‐based production is mostly in small amounts within a more controllable condition, it is uncertain how a lab‐efficient method may fit large‐scale production, especially considering the homogeneity and consistency of the products. Some extra control may be needed in the commercialized repairing or upcycling methods.
8) Similar to the above point, the recycled materials have been demonstrated only in the lab‐based devices, while the devices for the actual application are quite different. For example, the lab‐based coin cells normally contain 1–2 mg cm^−2^ cathode material, while it reaches 6–8 mg cm^−2^ for EV application, thus the distribution and adhesion of the cathode on the current collector shall be reconsidered during scaling‐up. Meanwhile, the lab‐based testing condition (e.g., constant current, ambient temperature, and humidity) is too idealized compared with real using conditions (variant current, ambient temperature and humidity, and strong vibration).
9) Following the above two points, a standard and wide‐accepted assessing system is needed to evaluate the economics of each repairing or upcycling method, as this is the intrinsic driving force and also the final goal of the repairing or upcycling spent LIBs.

## 6 Conclusion

The direct regeneration of spent LIBs cathode has only been prosperous in recent years. Compared with recycling valuable metals by conventional hydrometallurgical or pyrometallurgical methods, direct regeneration is considered as much energy saving and sustainable, as it relies mostly on fixing defects so the products can be returned directly to applications, whereas the hydrometallurgical or pyrometallurgical methods are based on completely structural destroy and further synthesis of cathodes are needed. The developed regeneration methods are diverse and have been executed on every kind of known commercial LIB cathodes, whereas their scaling‐up capability and commercialization are waiting to be verified. With techniques and policies gradually improved, it is expected that the market of recycling LIBs shall be standardized further so that more spent LIBs cathode will go to the direct regeneration route.

## Conflict of Interest

The authors declare no conflict of interest.

## Supporting information

Supporting Information
