# Supplementary material for: Direct Regenerating Cathode Materials from Spent Lithium‐Ion Batteries
Source: Adv Sci (Weinh). 2023 Nov 13;11(1):2304425. doi: 10.1002/advs.202304425 (PMC10767406; doi:10.1002/advs.202304425)
Supplement: Supplementary file 1 — Supporting Information [file ADVS-11-2304425-s001.pdf]

ADVANCED SCIENCE
Open Access

Supporting Information

for *Adv. Sci.*, DOI 10.1002/advs.202304425

Direct Regenerating Cathode Materials from Spent Lithium-Ion Batteries

Yuanqi Lan, Xinke Li, Guangmin Zhou, Wenjiao Yao, Hui-Ming Cheng* and Yongbing Tang**

Supplemental Information for

Direct regenerating cathode materials from spent lithium-ion batteries

Yuanqi Lan[#], Xinke Li[#], Guangmin Zhou, Wenjiao Yao*, Hui-Ming Cheng*, Yongbing Tang*

Yuanqi Lan, Xinke Li, Wenjiao Yao, Yongbing Tang
Advanced Energy Storage Technology Research Center, Shenzhen Institute of Advanced Technology, Chinese Academy of Sciences, Shenzhen, 518055, China
E-mail: wj.yao@siat.ac.cn; hm.cheng@siat.ac.cn; tangyb@siat.ac.cn

Yuanqi Lan, Yongbing Tang
Shenzhen College of Advanced Technology, University of Chinese Academy of Sciences, Shenzhen 518055, China

Xinke Li
Nano Science and Technology Institute, University of Science and Technology of China, Suzhou 215123, China

Guangmin Zhou
Shenzhen Geim Graphene Center, Tsinghua Shenzhen International Graduate School, Tsinghua University, Shenzhen 518055, China

Wenjiao Yao, Hui-Ming Cheng
Shenzhen Key Laboratory of Energy Materials for Carbon Neutrality, Shenzhen 518055, China

Hui-Ming Cheng
Faculty of Materials Science and Energy Engineering/Institute of Technology for Carbon Neutrality, Shenzhen Institute of Advanced Technology, Chinese Academy of Sciences Shenzhen 518055, P. R. China

Details evaluation information corresponding to Figure 4

Energy consumption evaluation for different methods						
**Methods**	**Heat-treatment temperature (0-10, 10 for high temperature treatment above 800℃, X)**	**Heat treatment time (0-1, 1 for more than 10 h, Y)**	**Extra electricity use (10 for yes, 0 for no, Z)**	**Score**	**Normalized score**	**Notes**
**High-temperature solid-state**	10	1	0	2	2.136752137	
**Hydrothermal/ ionothermal**	2	0.4	0	9.36	10	
**Molten salt thermodynamic**	4	0.4	0	8.72	9.316239316	
**Electrochemical**	0	0	10	8	8.547008547	
**Chemical relithiation**	1	0.8	0	9.36	10	
				Score=10-(X*Y*0.8+Z*0.2)		

Pollutant emission evaluation for different methods						
**Methods**	**Air pollutant**	**Sewage**	**Sludge**	**score**	**Normalized**	**Notes**

	emission (1-10, 10 for high emission, X)	emission (1-10, 10 for high emission, Y)	emission (1-10, 10 for high emission, Z)		score	
**High-temperature solid-state**	10	5	2	4.39	5.964673913	
**Hydrothermal/ ionothermal**	2	10	0	6.04	8.206521739	
**Molten salt thermodynamic**	1	2	5	7.36	10	
**Electrochemical**	5	10	5	3.4	4.619565217	Conventional electrochemical relithiation method
**Chemical relithiation**	5	10	0	4.5	6.114130435	
				Score=10-(X*0.33+Y*0.33+Z*0.33)		

Repair efficiency evaluation for different methods						
**Methods**	Relithiation	Crystal	Particle	Score	Normalized	

	efficiency (0-10, 10 for most effective, X)	recovery efficiency (0-10, 10 for most effective, Y)	recovery efficiency (0-10, 10 for most effective, Z)		score
**High-temperature solid-state**	8	10	10	9.1	10
**Hydrothermal/ ionothermal**	10	8	0	8.1	8.901098901
**Molten salt thermodynamic**	10	8	0	8.1	8.901098901
**Electrochemical**	8	4	0	5.4	5.934065934
**Chemical relithiation**	8	8	0	7.2	7.912087912
				Score= (X*0.45+Y*0.45+Z*0.1)	

Scalability evaluation for different methods						
**Methods**	**Could it be applied for doping (1 for yes, 0 for no, X)**	**Could it be applied for coating (1 for yes, 0 for no,Y)**	**The number of references mentioned about doping or coating in this article, Z**	**Score**	**Normalized score**	**Notes**
**High-temperature solid-state**	1	1	7	9.4	10	
**Hydrothermal/ ionothermal**	1	1	2	8.4	8.936170213	
**Molten salt thermodynamic**	0	0	0	0	0	evaluated with 2
**Electrochemical**	0	0	0	0	0	evaluated with 2
**Chemical relithiation**	0	0	0	0	0	evaluated with 2
				Score= X*4+Y*4+Z*0.2		

Processibility evaluation for different methods						
**Methods**	**The maturity for large scale production (1-10, 10 for perfectly mature, X)**	**The complexity of reactors (1-10, 10 for most complex,Y)**	**Simplicity（10 for simplest, Z）**	**Score**	**Normalized score**	**Notes**
**High-temperature solid-state**	10	8	10	9.24	10	
**Hydrothermal/ ionothermal**	6	4	8	6	6.493506494	
**Molten salt thermodynamic**	4	6	6	4	4.329004329	
**Electrochemical**	2	1	2	5.5	5.952380952	
**Chemical relithiation**	2	5	2	3.5	3.787878788	
				Score= X*0.33+Y*0.33+Z*0.33		

Cost evaluation for different methods						
**Methods**	**Energy consumption (1-10, 1 for highest energy consumption, X)**	**Chemical consumption (1-10, 1 for highest consumption,Y)**	**Waste emission handling (1-10, 1 for highest cost, Z)**	**Score**	**Normalized score**	**Notes**
**High-temperature solid-state**	2.136752137	8	4.964673913	4.7938282	6.59108536 2	
**Hydro thermal/ ionothermal**	10	6	7.206521739	7.2732	10	
**Melton salt thermodynamic**	9.316239316	2	9	6.163159	8.47379279 3	
**Electrochemical**	8.547008547	4	3.619565217	5.2625128	7.23548482 2	
**Chemical relithiation**	10	1	5.114130435	5.115	7.03266787 7	
	*Data from the normalized score of energy consumption evaluation for different methods.		*Data from the normalized score of pollutant emission evaluation for different methods	Score= X*0.33+Y*0. 33+Z*0.33		

References：

1. P. Xu, D. H. S. Tan, B. Jiao, H. Gao, X. Yu, Z. Chen, *Adv. Funct. Mater.* **2023**, 33, 2213168.

2. B. Raj, M. K. Sahoo, A. Nikoloski, P. Singh, S. Basu, M. Mohapatra, *Batteries Supercaps*. **2022**, 6, e202200418.

3. Y. Jin, T. Zhang, M. Zhang, *Adv. Energy Mater*. **2022**, 12, 2201526.

4. G. Harper, R. Sommerville, E. Kendrick, L. Driscoll, P. Slater, R. Stolkin, A. Walton, P. Christensen, O. Heidrich, S. Lambert, A. Abbott, K. Ryder, L. Gaines, P. Anderson, *Nature* **2019**, 575, 75.

5. P. Xu, Q. Dai, H. Gao, H. Liu, M. Zhang, M. Li, Y. Chen, K. An, Y. S. Meng, P. Liu, Y. Li, J. S. Spangenberger, L. Gaines, J. Lu, Z. Chen, *Joule* **2020**, 4, 2609.

6. J. Chen, Q. Li, J. Song, D. Song, L. Zhang, X. Shi, *Green Chem.* **2016**, 18, 2500.

7. G. Jiang, Y. Zhang, Q. Meng, Y. Zhang, P. Dong, M. Zhang, X. Yang, *ACS Sustain. Chem. Eng*. **2020**, 8, 18138.

8. L. Zhang, Z. Xu, Z. He, *ACS Sustain. Chem. Eng.* **2020**, 8, 11596.

9. Z. Fei, Y. Zhang, Q. Meng, P. Dong, Y. Li, J. Fei, H. Qi, J. Yan, *J. Hazard. Mater.* **2022**, 432, 128664.

10. T. Wang, H. Luo, Y. Bai, J. Li, I. Belharouak, S. Dai, *Adv. Energy Mater.* **2020**, 10, 2001204.
